# Astrocytic lactate dehydrogenase A regulates neuronal excitability and depressive-like behaviors through lactate homeostasis in mice

**DOI:** 10.1038/s41467-023-36209-5

**Published:** 2023-02-09

**Authors:** Shan Yao, Min-Dong Xu, Ying Wang, Shen-Ting Zhao, Jin Wang, Gui-Fu Chen, Wen-Bing Chen, Jian Liu, Guo-Bin Huang, Wen-Juan Sun, Yan-Yan Zhang, Huan-Li Hou, Lei Li, Xiang-Dong Sun

**Affiliations:** 1 Department of Neurology of the Second Affiliated Hospital, Institute of Neuroscience, Key Laboratory of Neurogenetics and Channelopathies of Guangdong Province and the Ministry of Education of China, School of Basic Medical Sciences, Guangzhou Medical University, Guangzhou, 510260 China; 2 Department of Physiology, School of Basic Medical Sciences, Guangzhou Medical University, Guangzhou, 511436 China; 3 Department of Physiology, Guangxi University of Science and Technology, Liuzhou, 545005 China; 4 Institute of Life Science, Nanchang University, Nanchang, 330031 China; 5 School of Life Science and Technology, ShanghaiTech University, Shanghai, 201210 China

**Keywords:** Molecular neuroscience, Cellular neuroscience

## Abstract

Alterations in energy metabolism are associated with depression. However, the role of glycolysis in the pathogenesis of depression and the underlying molecular mechanisms remain unexplored. Through an unbiased proteomic screen coupled with biochemical verifications, we show that the levels of glycolysis and lactate dehydrogenase A (LDHA), a glycolytic enzyme that catalyzes L-lactate production, are reduced in the dorsomedial prefrontal cortex (dmPFC) of stress-susceptible mice in chronic social defeat stress (CSDS) model. Conditional knockout of LDHA from the brain promotes depressive-like behaviors in both male and female mice, accompanied with reduced L-lactate levels and decreased neuronal excitability in the dmPFC. Moreover, these phenotypes could be duplicated by knockdown of LDHA in the dmPFC or specifically in astrocytes. In contrast, overexpression of LDHA reverses these phenotypic changes in CSDS-susceptible mice. Mechanistic studies demonstrate that L-lactate promotes neuronal excitability through monocarboxylic acid transporter 2 (MCT2) and by inhibiting large-conductance Ca^2+^-activated potassium (BK) channel. Together, these results reveal a role of LDHA in maintaining neuronal excitability to prevent depressive-like behaviors.

## Abstract (web summary)

The role of glycolysis in depression is unclear. Here the authors report a glycolytic deficit under social stress and demonstrate that astrocytic LDHA affects neuronal excitability and depressive-like behaviours via lactate homeostasis in mice.

## Introduction

Major depressive disorder (MDD) is one of the most common mental illnesses characterized by symptoms including anhedonia, social dysfunction, lack of motivation, and despair^1,2^. Although significant advances have been made in understanding the pathophysiology of MDD during the past decades, the exact underlying pathological mechanisms remain elusive, which profoundly limits the development of effective therapeutic agents.

Altered neural activities in specific brain regions in response to external stimuli, such as stress, have been considered a vital basis for MDD^3–6^. Numerous lines of evidence have indicated a critical role of the prefrontal cortex (PFC) in the pathophysiology of depression^7^. Brain imaging studies have reported decreased PFC volume and activity in depressed patients^8,9^. Consistently, elevating neural activity by optogenetic manipulation in the medial PFC (mPFC) could alleviate depressive-like behaviors^10^. Nevertheless, how the deficits of mPFC occur under depression is not well understood.

Considerable evidence has demonstrated that glucose metabolism is impaired in the PFC of depression patients and animal models^11–14^, which could be ameliorated by anti-depressant treatment^14^. These observations suggest an essential role of disturbed glucose metabolism in the pathogenesis of depression. Various factors, including defects in the electron transport chain, decreased mitochondrial potential, lower ATP levels, and oxidative damage, have been proposed to play detrimental roles in the development of depression^15–17^. Notably, human PFC is reported to be highly glycolytic at the resting state and further upregulates its glycolytic capacity upon brain activation^18,19^. Unexpectedly, few efforts have been devoted to dissecting the role of brain glycolysis, the first step of glucose metabolism, in depression pathogenesis.

During glycolysis, a glucose molecule is broken down into two pyruvate molecules by a series of enzymes, including hexokinase, enolase, pyruvate kinase, etc. Pyruvate either enters the mitochondria to be further oxidized into the tricarboxylic acid (TCA) cycle or is reversibly catalyzed by NADH(H^+^)-dependent lactate dehydrogenase (LDH) to L-lactate, which is not only an important energy substrate but also a signaling molecule in both peripheral organs and central nervous system^20,21^. Various studies have implicated that glycolysis-derived L-lactate in astrocytes can be exported to neurons and affects their survival and activity, which is required for memory consolidation and maintenance of the sleep-wake cycle^22–24^. These studies suggest a pivotal role of glycolysis and lactate shuttle in regulating brain physiology. In addition, several lines of evidence have demonstrated that peripheral administration of exogenous L-lactate exerts anti-depressant effects in both human patients and animal models^25–28^. While these observations suggest it as a promising therapeutic intervention for depression, a few questions remain unanswered: (a) Is endogenous lactate homeostasis altered in the brain under depression? (b) If yes, what cell type(s) are involved? and (c) how do these alterations contribute to the pathogenesis of depression?

Using a combination of proteomic, genetic, electrophysiological, and biochemical tools, we provide evidence that lactate dehydrogenase A (LDHA) in astrocytes, but not neurons, modulates neuronal excitability and depressive-like behaviors through maintaining lactate homeostasis in the dorsomedial PFC (dmPFC). We also investigated the underlying causal mechanisms of lactate regulation of depressive-like behaviors. We found that the large-conductance Ca^2+^-activated K^+^ (BK) channel mediates the beneficial effects of lactate on depressive-like phenotypes. Together, these results provide insight into the pathological mechanisms of depression.

## Results

### Chronic social-defeat stress causes reduced LDHA expression in the dmPFC

**Fig. 1:**
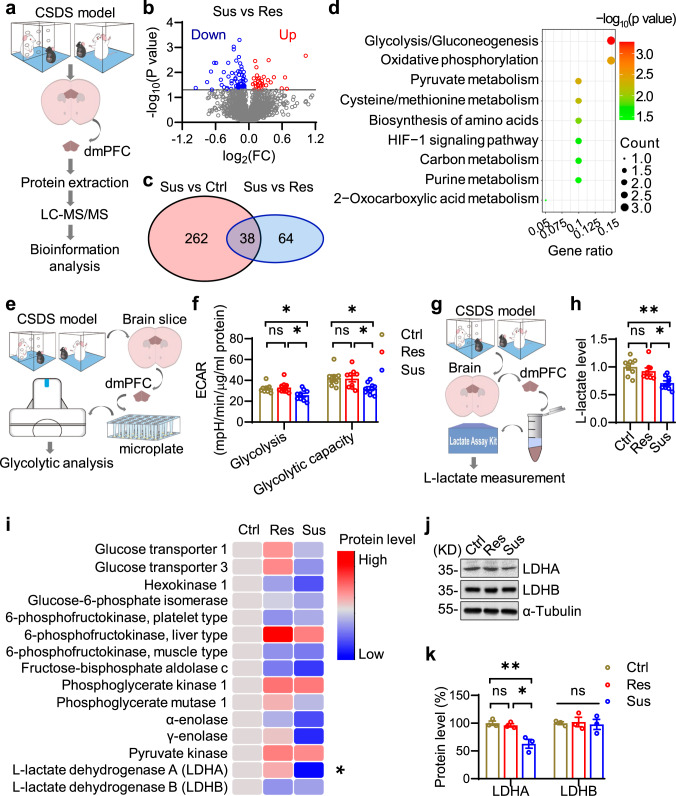
Compromised glycolysis and reduced LDHA expression in the dmPFC of social-defeated mice. **a** Schematic workflow of quantitative proteomic analysis. *n* = 5 mice per group. **b** Volcano plot of DEPs. Blue and red dots indicate significantly down- and up-regulated proteins, respectively. **c** Diagram of 38 DEPs between Sus vs ctrl and Sus vs Res comparisons. **d** Bubble plot of enriched KEGG pathways. **e** Schematic workflow of glycolytic analysis. **f** Quantitative glycolytic analysis. *n* = 8, 9 and 9 mice for Ctrl, Res and Sus groups, respectively. One-way ANOVA followed by Tukey’s post hoc test. For glycolysis, F_(2,23)_ = 5.604, *p* = 0.0104. Res vs Ctrl, *p* = 0.9356; Sus vs Res, *p* = 0.0141; Sus vs Ctrl, *p* = 0.0375; for glycolytic capacity, F_(2,23)_ = 4.61, *p* = 0.0207. Res vs Ctrl, *p* = 0.9862; Sus vs Res, *p* = 0.0431; Sus vs Ctrl, *p* = 0.0362. **g** Schematic workflow of L-lactate measurement. **h** Quantitative analysis. *n* = 8, 8, 9 mice for Ctrl, Res, Sus groups, respectively. One-way ANOVA followed by Tukey’s post hoc test. F_(2,22)_ = 9.071, *p* = 0.0013. Res vs Ctrl, *p* = 0.5909; Sus vs Res, *p* = 0.0158; Sus vs Ctrl, *p* = 0.0014. **i** Heatmap of glycolytic proteins. *n* = 5 mice per group. For LDHA analysis, One-way ANOVA followed by Tukey’s post hoc test. For LDHA, F_(2,12)_ = 9.549, *p* = 0.0033. Res vs Ctrl, *p* = 0.7816; Sus vs Res, *p* = 0.004; Sus vs Ctrl, *p* = 0.0135. **j** Representative Western blot images. **k** Quantitative analysis of data in (**j**). *n* = 3 mice per group. One-way ANOVA followed by Tukey’s post hoc test. For LDHA, F_(2,6)_ = 14.17, *p* = 0.0053. Res vs Ctrl, *p* = 0.8673; Sus vs Res, *p* = 0.0117; Sus vs Ctrl, *p* = 0.0068; for LDHB, F_(2,6)_ = 0.088, *p* = 0.9169. Data were shown as mean ± SEM. *p < 0.05, **p < 0.01; ns, no significant difference. Source data are provided as a Source Data file.

We utilized the chronic social-defeat stress model (CSDS) to induce depressive-like phenotypes in mice (Supplementary Fig. 1a). Mice with substantial social avoidance (Refer to as Sus mice) had a longer duration of immobility in the forced swimming test (FST) and less sucrose consumption in the sucrose preference test (SPT) in comparison to mice resistant to social avoidance (Refer to as Res mice) and those without exposure to social defeat (Refer to as Ctrl mice) (Supplementary Fig. 1b–d). In addition, the serum corticosterone levels in both Res and Sus mice were elevated compared with those in Ctrl mice (Supplementary Fig. 1e), in line with a previous report^29^. An unbiased quantitative proteomic screening was conducted in the dmPFC (Fig. 1a), a critical brain region for depression pathogenesis^7^. The results showed that, out of total 3513 identified proteins, 49 and 251 proteins were up- and down-regulated, respectively, in Sus mice compared with Ctrl mice (Supplementary Fig. 2a); 32 and 70 proteins were up- and down-regulated in Sus mice in comparison to Res mice (Fig. 1b). Out of all the differentially expressed proteins (DEPs), 38 were identified in both comparisons (Fig. 1c and supplementary Fig. 2b). Gene Ontology (GO) analysis of these DEPs highlighted various metabolic processes including glucose metabolism and ATP metabolic process (Supplementary Fig. 2c), in line with previous reports^15,16^. Noticeably, Kyoto Encyclopedia of Genes and Genomes (KEGG) analysis indicated that glycolysis/gluconeogenesis might be involved in depression (Fig. 1d).

To examine whether the glycolytic activity in dmPFC is altered under a depression state, we measured extracellular acidification rate (ECAR) using a Seahorse Analyzer (Fig. 1e). The ECAR levels in the dmPFC of Sus mice following both glucose and oligomycin injections were significantly lower than those in Ctrl and Res mice (Fig. 1f), indicating compromised glycolysis and glycolytic capacity. In agreement, the extracellular L-lactate levels were significantly lower in the dmPFC of Sus mice than those in Ctrl and Res mice (Fig. 1g, h). To investigate the underlying molecular mechanisms, we compared the levels of major glycolytic enzymes from proteomic data. Notably, the LDHA level in Sus mice was decreased when compared with that in Ctrl and Res mice (Fig. 1i). This result was confirmed by immunoblotting analysis showing an attenuated level of LDHA but not LDHB in Sus mice compared with that in Ctrl and Res mice (Fig. 1j, k). Altogether, these observations demonstrate that the extent of glycolysis and LDHA expression are reduced in the dmPFC of social-defeated mice.

### *Ldha* deletion in the dmPFC promotes depressive-like behaviors and reduces neuronal excitability

**Fig. 2:**
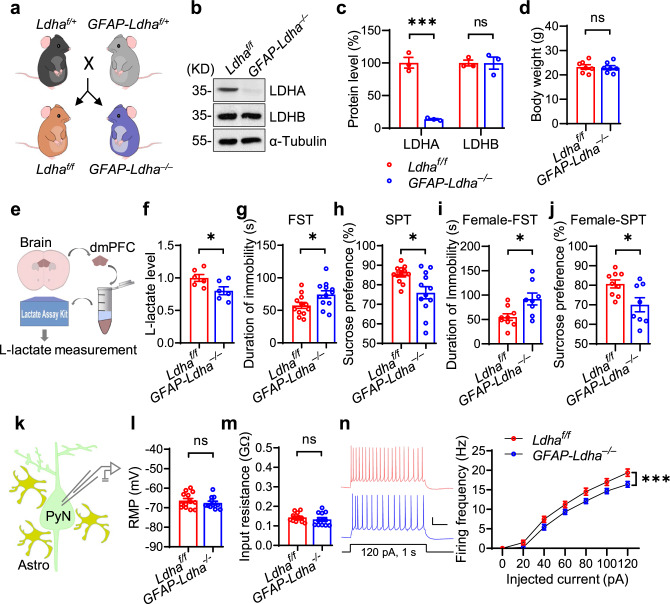
*Ldha* deletion in the brain causes depressive-like behaviors and reduces neuronal excitability. **a** Breeding strategy. **b** Representative Western blot images. **c** Quantitative analysis of data in (**b**). *n* = 3 mice per genotype. Student’s *t*-test, for LDHA, t_(4)_ = 10.49, *p* = 0.0005; for LDHB, t_(4)_ =  0.0144, *p* = 0.9892. **d** Similar bodyweight between *Ldha*^*f/f*^ and *GFAP-Ldha*^*–/–*^ mice. *n* = 7 mice per genotype. Student’s *t*-test, t_(12)_ = 0.2446, *p* = 0.8109. **e**, **f** Reduced L-lactate levels in the dmPFC of *GFAP-Ldha*^*–/–*^ mice. *n* = 6 mice per genotype. Student’s *t*-test, t_(10)_ = 2.435, *p* = 0.0351. **g**, **h** Increased immobility duration (**g**) and decreased sucrose preference (**h**) in *GFAP-Ldha*^*–/–*^ mice. *n* = 12 mice per genotype. Student’s *t*-test, for FST, t_(22)_ = 2.32, *p* = 0.03; for SPT, t_(22)_ = 2.804, *p* = 0.0103. **i**, **j** Increased immobility duration (**i**) and decreased sucrose preference (**j**) in female *GFAP-Ldha*^*–/–*^ mice. *n* = 9 and 8 female mice for *Ldha*^*f/f*^ and *GFAP-Ldha*^*–/–*^ groups, respectively. Student’s *t*-test, for FST, t_(15) _= 2.729, *p* = 0.0155; for SPT, t_(15)_ = 2.663, *p* = 0.0177. **k** Schematic diagram of recording in dmPFC region. PyN pyramidal neuron, Astro astrocyte. **l** Similar RMP between *Ldha*^*f/f*^ and *GFAP-Ldha*^*–/–*^ pyramidal neurons. *n* = 13 and 12 neurons from 3 *Ldha*^*f/f*^ and 3 *GFAP-Ldha*^*–/–*^ mice, respectively. Student’s *t*-test, t_(23)_ = 0.8311, *p* = 0.4145. **m** Comparable input resistance between *Ldha*^*f/f*^ and *GFAP-Ldha*^*–/–*^ pyramidal neurons. *n* = 13 and 12 neurons from 3 *Ldha*^*f/f*^ and 3 *GFAP-Ldha*^*–/–*^ mice, respectively. Student’s *t*-test, t_(23)_ =  0.9388, *p* = 0.3576. **n** Compromised neuronal excitability. Left, representative firing traces. Scale bars: 0.2 s, 20 mV. Right, quantitative data. *n* = 13 and 12 neurons from 3 *Ldha*^*f/f*^ and 3 *GFAP-Ldha*^*–/–*^ mice, respectively. Two-way ANOVA, gene effect, F_(1,161)_ = 25.09, *p* = 0.0000. Data were shown as mean ± SEM. *p < 0.05, ***p < 0.001; ns, no significant difference. Source data are provided as a Source Data file.

To specifically investigate the role of LDHA in the brain, we generated *Ldha* conditional knockout mice by crossing *Ldha-loxp* (*Ldha*^*f/f*^) mice with *GFAP-Cre* mice (Fig. 2a, Supplementary Fig. 3a–c). This Cre line expresses Cre recombinase under the promoter of GFAP, whose activity is present in neural progenitor cells, which give rise to neurons and astrocytes^30,31^. Western blot data showed that the LDHA level was dramatically decreased in the dmPFC of *Ldha* mutant mice (Refer to as *GFAP-Ldha*^*–/–*^) (Fig. 2b, c). Body weight and densities of neurons and astrocytes in the dmPFC of *Ldha*^*f/f*^ and *GFAP-Ldha*^*–/–*^ mice were similar (Fig. 2d; Supplementary Fig. 3d, e), suggesting a limited role of LDHA in gross brain development. However, extracellular L-lactate levels were decreased in the dmPFC of *GFAP-Ldha*^*–/–*^ mice (Fig. 2e, f). Behaviorally, *GFAP-Ldha*^*–/–*^ mice showed no differences in traveled distance, duration in the center, and the number of entries to the center in the open field test, and no difference in the time and frequency in the open arms in the elevated plus-maze test when compared to *Ldha*^*f/f*^ mice (Supplementary Fig. 4a–g). These observations suggest that the locomotor activity and anxiety level are not affected by *Ldha* deletion. However, *GFAP-Ldha*^*–/–*^ mice exhibited a longer duration of immobility in the FST and a lower ratio of sucrose preference in the SPT compared to *Ldha*^*f/f*^ mice (Fig. 2g, h). In addition, the success rate of climbing over the barrier to achieve a food reward was decreased in the *GFAP-Ldha*^*–/–*^ mice in the T-maze barrier choice task (Supplementary Fig. 4h–k), suggesting an impaired reward function. Together, these results indicate a critical role of LDHA in the regulation of depressive-like behaviors. Note that *Ldha* deletion in female mice also induced depressive-like behaviors (Fig. 2i, j), indicating that the effects of LDHA on depression phenotypes are gender-independent. To explore the underlying cellular mechanisms, we recorded pyramidal neurons in the dmPFC in a whole-cell configuration (Fig. 2k). The membrane potential and input resistance were not changed by *Ldha* deletion (Fig. 2i, m). While the frequency and amplitude of miniature excitatory postsynaptic currents (mEPSCs) as well as those of miniature inhibitory postsynaptic currents (mIPSCs) were not altered (Supplementary Fig. 5), the neuronal firing frequencies in response to depolarizing current injections were significantly decreased in *Ldha* mutant mice (Fig. 2n), These data demonstrate an important role of LDHA in maintaining neuronal excitability in the dmPFC.

**Fig. 3:**
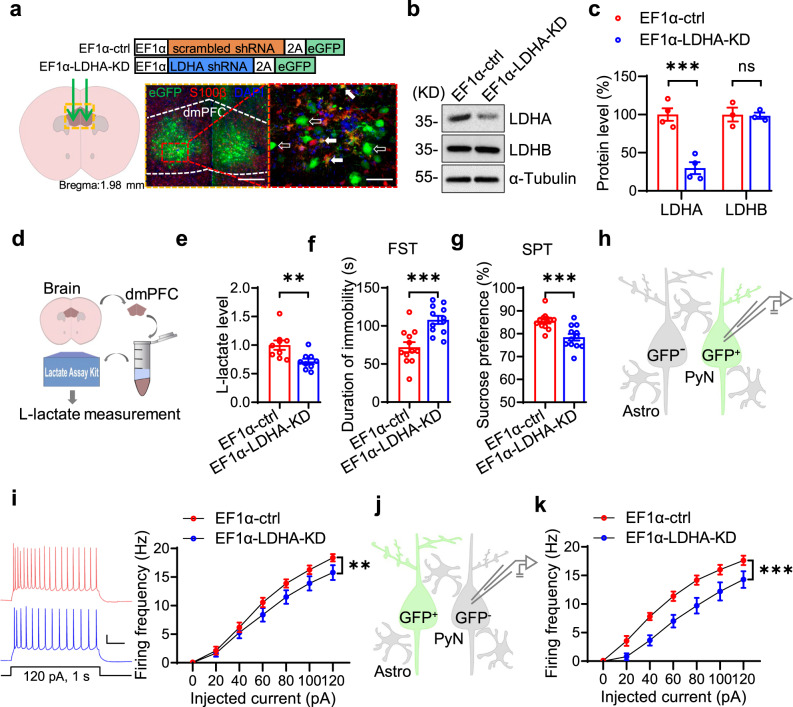
Knockdown of LDHA in the dmPFC leads to depressive-like behaviors. **a** Top, schematics of AAV vectors; bottom, illustration of bilateral viral injections in dmPFC region (stained with antibody against S100β and DAPI). White solid arrows denote GFP^+^ and S100β^+^ cells; white hollow arrows denote GFP^+^ and S100β^−^ cells. Scale bars: left, 200 µm; right, 50 µm. **b** Representative Western blot images. **c** Quantitative analysis of data in (**b**). *n* = 4 and 3 mice per group for LDHA and LDHB, respectively. Student’s *t*-test, for LDHA, t_(6)_ =  6.197, *p* = 0.0008; for LDHB, t_(4)_ = 0.1517, *p* = 0.8868. **d** Schematic workflow of L-lactate measurement. **e** Reduced L-lactate levels in dmPFC of EF1α-LDHA-KD mice. *n* = 9 mice per group. Student’s *t*-test, t_(16)_ = 2.993, *p* = 0.0086. **f** Increased immobility duration in EF1α-LDHA-KD mice in FST test. *n* = 12 mice per genotype. Student’s *t*-test, t_(22)_ = 4.26, *p* = 0.0003. **g** Decreased sucrose preference in EF1α-LDHA-KD mice in SPT test. *n* = 12 mice per genotype. Student’s *t*-test, t_(22)_ = 3.937, *p* = 0.0007. **h** Schematic diagram of recording of GFP^+^ neurons in dmPFC region. PyN pyramidal neuron, Astro astrocyte. **i** Compromised neuronal excitability of GFP^+^ neurons in dmPFC of EF1α-LDHA-KD mice. Left, representative firing traces. Scale bars: 0.2 s, 20 mV. Right, quantitative data. *n* = 16 and 18 neurons from 5 EF1α-ctrl and 5 EF1α-LDHA-KD mice, respectively. Two-way ANOVA, gene effect, F_(1,224)_ = 10.62, *p* = 0.0013. **j** schematic diagram of recording of GFP^−^ neurons in dmPFC region. **k** Compromised neuronal excitability of GFP^−^ neurons in dmPFC of EF1α-LDHA-KD mice. Left, representative firing traces. Scale bars: 0.2 s, 20 mV. Right, quantitative data. *n* = 11 and 14 neurons from 5 EF1α-ctrl and 5 EF1α-LDHA-KD mice. Two-way ANOVA, gene effect, F_(1,161)_ = 36.83, *p* = 0.0000. Data were shown as mean ± SEM. **p < 0.01, ***p < 0.001; ns, no significant difference. Source data are provided as a Source Data file.

To determine whether LDHA in the dmPFC is sufficient to regulate depressive-like behaviors, we knocked down LDHA by bilateral injection of adeno-associated virus (AAV) vectors expressing a short hairpin RNA (shRNA) that specifically targets the LDHA transcript (EF1α-LDHA-shRNA) into the dmPFC (Fig. 3a). Western blot analysis showed reduced LDHA levels in the dmPFC of EF1α-LDHA-shRNA-injected mice (Refer to as EF1α-LDHA-KD) compared with control mice (EF1α-ctrl) (Fig. 3b, c). We found that the L-lactate levels were decreased in the dmPFC of EF1α-LDHA-KD mice (Fig. 3d, e). Behaviorally, EF1α-LDHA-KD mice showed increased duration of immobility in the FST and decreased sucrose preference in the SPT compared with EF1α-ctrl mice (Fig. 3f, g). These results indicate that LDHA in the dmPFC is required for the maintenance of endogenous L-lactate homeostasis and resistance to depressive-like behaviors. We then performed electrophysiological recording to examine the effects of *Ldha* deletion on neuronal excitability. In light that both neurons and non-neuronal cells in the dmPFC were infected by the AAV vectors (Fig. 3a), and neuronal excitability could be affected in a cell-autonomous or non-cell-autonomous manner^32,33^, we therefore recorded both virus-infected (GFP^+^) and non-infected (GFP^−^) neurons. The results showed that the firing frequencies of both virus-infected (GFP^+^) and non-infected (GFP^−^) neurons from EF1α-LDHA-KD mice were reduced compared with those from control mice (Fig. 3h–k). These observations suggest that *Ldha* deletion decreases neuronal excitability in a non-cell-autonomous manner.

### Astrocytic LDHA is required for modulating neuronal excitability and depressive-like behaviors

**Fig. 4:**
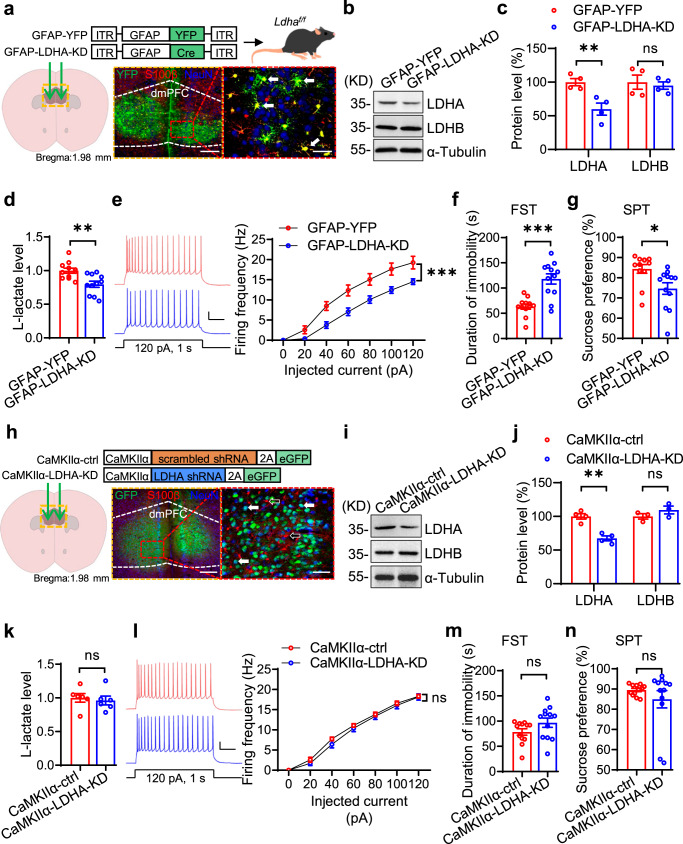
Astrocytic LDHA is required for modulation of neuronal excitability and depressive-like behaviors. **a** Illustration of viral injections. White arrows denote YFP^+^, S100β^+^ and NeuN^−^ neurons. Scale bars, 200 µm and 50 µm. **b**, **c** Representative Western blot images (**b**) and quantification (**c**). *n* = 4 mice per group. Student’s *t*-test, for LDHA, t_(6)_ = 3.83, *p* = 0.0087; for LDHB, t_(6)_ = 0.4299, *p* = 0.6823. **d** Decreased L-lactate levels. *n* = 11 mice per group. Student’s *t*-test, t_(20)_ = 3.408, *p* = 0.0028. **e** Compromised neuronal excitability. Left, representative firing traces. Scale bars: 0.2 s, 20 mV. Right, quantitative data. *n* = 12 and 13 neurons from 3 GFAP-YFP and 3 GFAP-Cre mice, respectively. Two-way ANOVA, gene effect, F_(1,161)_ = 53.32, *p* = 0.0000. **f**, **g** Increased immobility duration (**f**) and decreased sucrose preference (**g**). *n* = 12 mice per group. Student’s *t*-test, for FST, t_(22)_ = 4.804, *p* = 0.0001; for SPT, t_(22)_ = 2.722, *p* = 0.0125. **h** Illustration of viral injections. White solid arrows denote both GFP^+^ and NeuN^+^ cells; white hollow arrows denote GFP^−^ and S100β^+^ cells. Scale bars, 200 µm and 50 µm. **i**, **j** Representative Western blot images (**i**) and quantification (**j**). *n* = 4 and 3 mice per group for LDHA and LDHB, respectively. Student’s *t*-test, for LDHA, t_(6)_ = 5.957, *p* = 0.001; for LDHB, t_(4)_ = 1.324, *p* = 0.2561. **k** Similar L-lactate levels. *n* = 6 mice per group. Student’s *t*-test, t_(10)_ = 0.4331, *p* = 0.6741. **l** Comparable neuronal excitability. Left, representative firing traces. Scale bars: 0.2 s, 20 mV. Right, quantitative data. *n* = 12 and 15 neurons from 3 CaMKIIα-ctrl and 3 CaMKIIα-LDHA-KD mice, respectively. Two-way ANOVA, gene effect, F_(1,175)_ = 2.675, *p* = 0.1038. **m**, **n** Comparable immobility duration (**m**) and sucrose preference (**n**). *n* = 12 mice per group. Student’s *t*-test, for FST, t_(22)_ = 1.634, *p* = 0.1164; for SPT, t_(22)_ =  1.053, *p* = 0.3036. Data were shown as mean ± SEM. *p < 0.05, **p < 0.01, ***p < 0.001; ns, no significant difference. Source data are provided as a Source Data file.

Previous reports have suggested a preferential expression of LDHA in astrocytes of the brain^21,34^. However, the data from the RNAseq database indicate that LDHA is also expressed in neurons (Refer to website: www.brainrnaseq.org). This notion was confirmed by our western blotting results (Supplementary Fig. 6). To determine whether astrocytic LDHA is required for regulating depressive-like behaviors, we injected AAV-GFAP-Cre virus into the dmPFC of *Ldha*^*f/f*^ mice to specifically delete *Ldha* from astrocytes (Fig. 4a). The specificity of astrocyte infection by GFAP-YFP and GFAP-Cre viruses was validated by injecting them into the dmPFC of C57 mice and *Rosa::LSL-tdTomato* (*td*) reporter mice, respectively. Quantitative analysis revealed the presence of astrocytic marker-S100β in 91.43% of YFP^+^ cells, whereas only 2.37% of YFP^+^ cells were positive for NeuN (a neuronal marker) (Supplementary Fig. 7a, b). More importantly, tdTomato^+^ cells were hardly labeled by NeuN (7.40%, Supplementary Fig. 7c, d), indicating that the Cre activity is specific to astrocytes. Western blot results showed decreased LDHA level in GFAP-Cre virus-injected *Ldha*^*f/f*^ mice (GFAP-LDHA-KD) compared with control virus-injected *Ldha*^*f/f*^ mice (GFAP-YFP) (Fig. 4b, c). We found that both L-lactate levels and neuronal firing frequencies in the dmPFC were reduced in GFAP-LDHA-KD mice (Fig. 4d, e). Moreover, GFAP-LDHA-KD mice displayed an increased duration of immobility in the FST, decreased sucrose preference in the SPT, and impaired social behavior in the social interaction test compared with GFAP-YFP mice (Fig. 4f, g), indicating the emergence of depressive-like behaviors. In contrast, *Ldha* deletion in neurons by injection of virus expressing CaMKIIα-LDHA-shRNA into the dmPFC failed to change lactate level, neuronal excitability, or depressive-like behaviors (Fig. 4h–n; Supplementary Fig. 8). Together, these findings suggest that LDHA in astrocytes, but not excitatory neurons, is critical for the regulation of L-lactate production, neuronal excitability, and depressive-like behaviors.

### LDHA overexpression in the dmPFC alleviates depressive-like behaviors in social-defeated mice

**Fig. 5:**
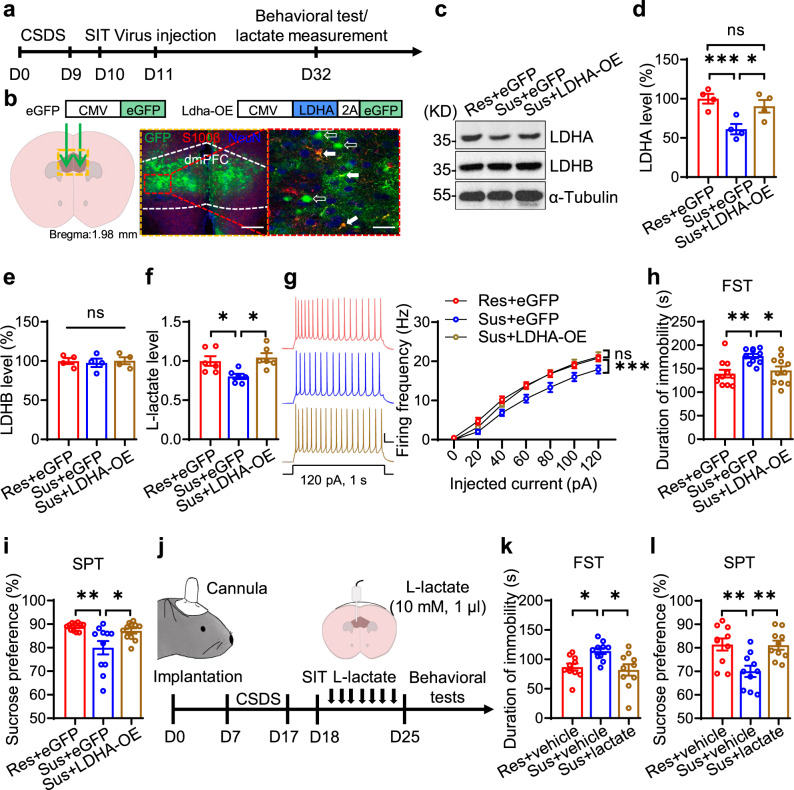
LDHA overexpression and L-lactate infusion alleviate depressive-like behaviors of CSDS-susceptible mice. **a** Experimental time scheme. SIT social interaction test. **b** Illustration of viral injections. Solid arrows, GFP^+^ and S100β^+^; hollow arrows, GFP^+^ and NeuN^+^. Scale bars: 200 µm, 50 µm. **c**–**e** Representative Western blot images (**c**) and quantification (**d**, **e**). *n* = 4 mice per group. One-way ANOVA followed by Tukey’s post hoc test. for LDHA, F_(2,9)_ = 8.304, *p* = 0.009. Sus+eGFP vs Res+eGFP, *p* = 0.009; Sus+LDHA-OE vs Sus+eGFP, *p* = 0.0392; Sus+LDHA-OE vs Res+eGFP, *p* = 0.6171. For LDHB, F_(2,9)_ = 0.102, *p* = 0.9041. **f** Restored L-lactate levels. *n* = 6 mice per group. One-way ANOVA followed by Tukey’s post hoc test. F_(2,15)_ = 6.191, *p* = 0.011. Sus+eGFP vs Res+eGFP, *p* = 0.0415; Sus+LDHA-OE vs Sus+eGFP, *p* = 0.0125. **g** Restored neuronal excitability. Left, representative firing traces. Scale bars: 0.2 s, 20 mV. Right, quantitative data. *n* = 15, 15, 16 neurons from 4 Res+eGFP, 4 Sus+eGFP, 4 Sus+LDHA-OE mice, respectively. Two-way ANOVA, for Sus+LDHA-OE vs Sus+eGFP, gene effect, F_(1,203)_ = 26.68, *p* = 0.0000; for Sus+LDHA-OE vs Res+eGFP, gene effect, F_(1,203)_ = 0.7911, *p* = 0.3748. **h**, **i** Reversed immobility duration (**h**) and sucrose preference (**i**). *n* = 11 mice per group. One-way ANOVA followed by Tukey’s post hoc test. For FST, F_(2,30)_ = 7.3, *p* = 0.0026. Sus+eGFP vs Res+eGFP, *p* = 0.0033; Sus+LDHA-OE vs Sus+eGFP, *p* = 0.0166. For SPT, F_(2,23)_ = 6.863, *p* = 0.0035. Sus+eGFP vs Res+eGFP, *p* = 0.0041; Sus+LDHA-OE vs Sus+eGFP, *p* = 0.0233. **j** Experimental time scheme. **k**, **l** Reversed immobility duration (**k**) and sucrose preference (**l**). *n* = 10 mice per group. One-way ANOVA followed by Tukey’s post hoc test. For FST, F_(2,27)_ = 5.748, *p* = 0.0083. Sus+vehicle vs Res+vehicle, *p* = 0.0332; Sus+L-lactate vs Sus+vehicle, *p* = 0.0108. For SPT, F_(2,27)_ = 7.805, *p* = 0.0021. Sus+vehicle vs Res+vehicle, *p* = 0.0049; Sus+L-lactate vs Sus+vehicle, *p* = 0.0062. Data were shown as mean ± SEM. *p < 0.05, **p < 0.01, ***p < 0.001; ns, no significant difference. Source data are provided as a Source Data file.

To determine whether LDHA overexpression in the dmPFC is sufficient to increase neuronal excitability and alleviate depressive-like phenotypes of social-defeated mice, we injected AAVs expressing LDHA (refer to as LDHA-OE virus) or eGFP into the dmPFC of Sus and Res mice (Fig. 5a, b). Three weeks later, we observed that the LDHA levels in the dmPFC of Sus mice injected with LDHA-OE virus (Refer to as Sus+LDHA-OE mice) were increased compared with those in Sus mice injected with eGFP virus (Refer to as Sus+eGFP mice) and were similar to those in Res mice injected with eGFP virus (Refer to as Res+eGFP mice). There was no difference in LDHB level among these mice (Fig. 5c–e). Consistently, the levels of L-lactate were also elevated in Sus+LDHA-OE mice compared with Sus+eGFP mice (Fig. 5f). Electrophysiological results indicated that LDHA overexpression increased the firing frequencies of dmPFC neurons in Sus+LDHA-OE mice, to a level comparable to Res+eGFP mice (Fig. 5g). Behaviorally, LDHA overexpression profoundly reduced the duration of immobility in the FST and increased the ratio of sucrose preference in the SPT (Fig. 5h, i). These results indicate that LDHA overexpression in the dmPFC is sufficient to restore L-lactate homeostasis, increase neuronal excitability, and reduce depressive-like phenotypes.

To investigate whether the depressive-like behaviors of social-defeated mice could be rescued by increasing L-lactate levels in the dmPFC, we conducted chronic unilateral injection of L-lactate (10 mM, 1 µl) into dmPFC each day for one week. A representative image presenting the appropriate infusion site was shown in Supplementary Fig. 9a. Note that a single infusion of L-lactate into the dmPFC increased the extracellular L-lactate level in a dose and time-dependent manner (Supplementary Fig. 9b). Behavioral analysis showed a shorter duration of immobility and higher sucrose preference in L-lactate-injected Sus mice (Sus+L-lactate) in comparison to vehicle-injected Sus mice (Sus+vehicle) (Fig. 5j–l). These results implicate a therapeutic effect of L-lactate on depressive phenotypes, in line with previous studies^25,26^.

### MCT2 in dmPFC pyramidal neurons mediates the anti-depressive effects of L-lactate

Based on the above results, it is plausible to hypothesize that LDHA deficiency disrupts L-lactate homeostasis, which impairs neuronal excitability and eventually leads to depressive-like phenotypes. It has been reported that hydroxycarboxylic acid receptor 1 (HCAR1), a G-protein-coupled receptor for L-lactate, modulates neuronal activity^35,36^. To determine whether HCAR1 participate in the CSDS-induced depressive-like phenotypes, we generated AAVs expressing HCAR1-shRNA and injected them into the dmPFC of C57 wild-type mice (CAG-HCAR1-KD) (Supplementary Fig. 10a). We detected a reduced mRNA level of *Hcar1* through RT-PCR analysis (Supplementary Fig. 10b). However, membrane potential, input resistance, and firing frequencies were not different between CAG-HCAR1-KD and scrambled shRNA virus-injected mice (CAG-ctrl) (Supplementary Fig. 10c–f), indicating a limited role of HCAR1 in the regulation of dmPFC neuronal excitability. Moreover, the duration of immobility and the sucrose preference ratio were also comparable between CAG-HCAR1-KD- and CAG-ctrl mice (Supplementary Fig. 10g, h). Collectively, these data suggest that HCAR1 is dispensable for L-lactate’s anti-depressive effects.

**Fig. 6:**
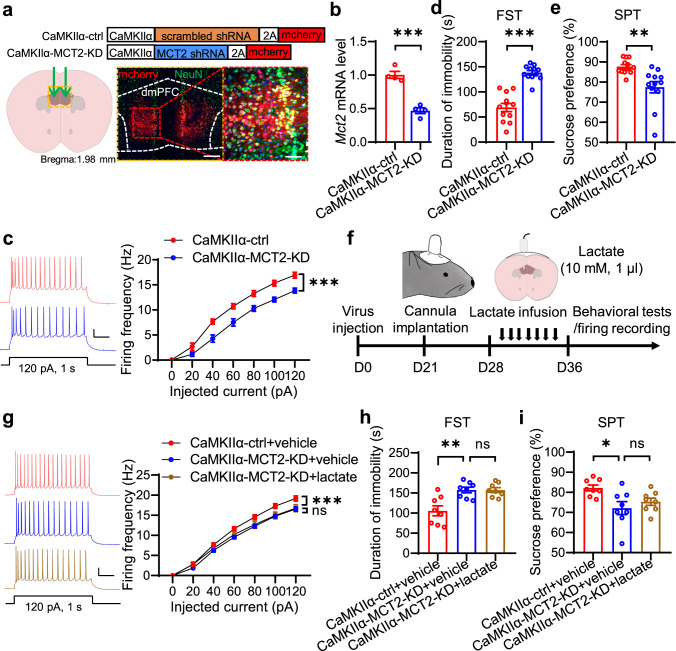
Neuronal MCT2 in the dmPFC mediates the anti-depressive effects of L-lactate. **a** Illustration of viral injections. Scale bars: left, 150 µm; right, 50 µm. **b** Reduced *Mct2* mRNA level by shRNA against Mct2. *n* = 4 mice per group. Student’s *t*-test, t_(6)_ = 7.928, *p* = 0.0002. **c** Compromised neuronal excitability of neurons in dmPFC of CaMKIIα-MCT2-KD mice. Left, representative firing traces. Scale bars: 0.2 s, 20 mV. Right, quantitative data. *n* = 15 and 14 neurons from 4 CaMKIIα-ctrl and 4 CaMKIIα-MCT2-KD mice. Two-way ANOVA, gene effect, F_(1,189)_ = 75.12, *p* = 0.0000. **d**, **e** Increased immobility duration (**d**) and decreased sucrose preference (**e**) of CaMKIIα-MCT2-KD mice. *n* = 12 mice per group. Student’s *t*-test, for FST, t_(22)_ = 7.514, *p* = 0.0000; for SPT, t_(22)_ = 3.417, *p* = 0.0025. **f** Experimental time scheme. **g** Diminished effect of L-lactate on neuronal excitability by MCT2 knockdown. Left, representative firing traces. Scale bars: 0.2 s, 20 mV. Right, quantitative data. *n* = 18, 22 and 19 neurons from 4 CaMKIIα-ctrl+vehicle, 4 CaMKIIα-MCT2-KD + vehicle mice and 4 CaMKIIα-MCT2-KD + L-lactate mice. Two-way ANOVA, for CaMKIIα-MCT2-KD + vehicle vs CaMKIIα-ctrl+vehicle, gene effect, F_(1,266)_ = 53.06, *p* = 0.0000; for CaMKIIα-MCT2-KD + L-lactate vs CaMKIIα-ctrl+vehicle, gene effect, F_(1,273)_ = 1.974, *p* = 0.1611. **h**, **i** Diminished effect of L-lactate on the immobility duration (**h**) and sucrose preference (**i**) by MCT2 knockdown. *n* = 8 mice per group. One-way ANOVA followed by Tukey’s post hoc test. For FST, F_(2,21)_ = 8.067, *p* = 0.0025. CaMKIIα-MCT2-KD + vehicle vs CaMKIIα-ctrl+vehicle, *p* = 0.0051; CaMKIIα-MCT2-KD + L-lactate vs CaMKIIα-ctrl+vehicle, *p* = 0.9853. For SPT, F_(2,21) _= 5.041, *p* = 0.0163. CaMKIIα-MCT2-KD + vehicle vs CaMKIIα-ctrl+vehicle, *p* = 0.0142; CaMKIIα-MCT2-KD + L-lactate vs CaMKIIα-ctrl+vehicle, *p* = 0.5953. Data were shown as mean ± SEM. *p < 0.05, **p < 0.01, ***p < 0.001; ns, no significant difference. Source data are provided as a Source Data file.

Next, we investigated the role of MCT2, an L-lactate transporter, which is mainly expressed in the neurons^37^. AAVs expressing MCT2-shRNA driven by the CaMKIIα promoter were injected into the dmPFC (Fig. 6a). RT-PCR analysis revealed dramatically decreased *Mct2* mRNA level in the dmPFC of MCT2-shRNA virus-injected mice (CaMKIIα-MCT2-KD) compared with scrambled shRNA virus-injected mice (CaMKIIα-ctrl) (Fig. 6b). CaMKIIα-MCT2-KD mice exhibited reduced firing frequencies in the dmPFC, increased duration of immobility in the FST, and decreased sucrose preference in the SPT (Fig. 6c–e), demonstrating a pivotal role of MCT2 in the modulation of neuronal excitability and depressive-like behaviors. In addition, L-lactate infusion into the dmPFC failed to rescue the neuronal firing and depressive-like phenotypes in CaMKIIα-MCT2-KD mice (Fig. 6f–i). Together, these results demonstrate that MCT2 in pyramidal neurons of dmPFC is required for the anti-depressive effects of L-lactate.

### L-lactate increases neuronal excitability via inhibiting fast after-hyperpolarization

**Fig. 7:**
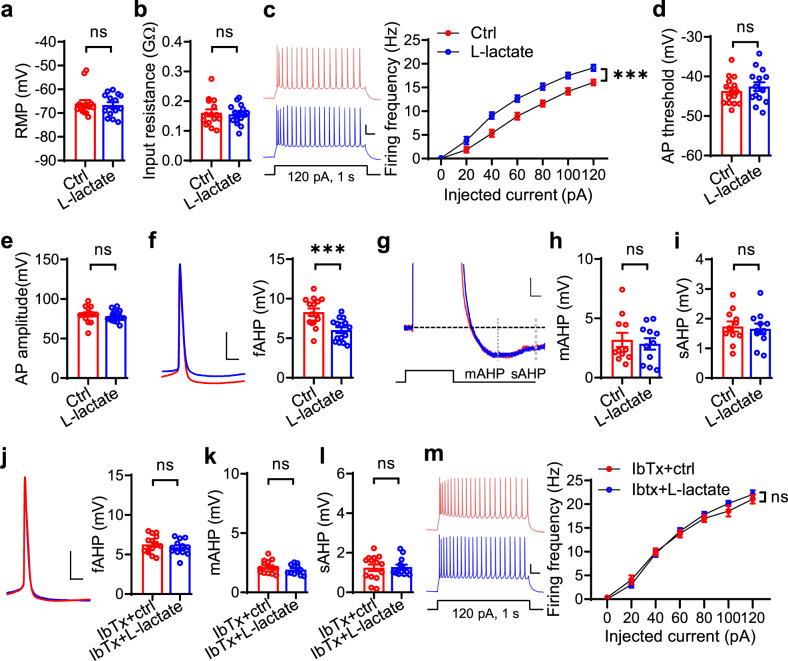
L-lactate increases neuronal excitability through downregulation of fAHP. **a**, **b** Little effect of L-lactate on RMP (**a**) and input resistance (**b**). *n* = 15 neurons from 5 mice per group. Student’s *t*-test, for RMP, t_(28)_ = 0.3566, *p* = 0.7241; for input resistance, t_(28)_ = 0.313, *p* = 0.7566. **c**–**e** L-lactate increases neuronal excitability (**c**) without effect on the threshold (**d**) and amplitude (**e**) of action potential. Left, representative firing traces. Scale bars: 0.1 s, 20 mV. *n* = 15 neurons from 5 mice per group. For excitability, two-way ANOVA, gene effect, F_(1,196)_ = 65.08, *p* = 0.0000; for threshold, Student’s *t*-test, t_(28)_ = 0.8113, *p* = 0.4241; for amplitude, t_(28) _= 0.6411, *p* = 0.5266. **f** Reduced fAHP. *n* = 15 neurons from 5 mice per group. Left, representative firing traces. Scale bars: 10 s, 20 mV. Right, quantitative data. Student’s *t*-test, t_(28) _= 3.882, *p* = 0.0006. **g** Representative AHP traces. Scale bars: 40 ms, 2 mV. **h-i** Minimal effect of L-lactate on mAHP (**h**) and sAHP (**i**). *n* = 11 neurons from 4 male mice per group. Student’s *t*-test, for mAHP, t_(20)_ = 0.4404, *p* = 0.6644; for sAHP, Student’s *t*-test, t_(20)_ = 0.313, *p* = 0.7575. **j**–**l** Diminished effect of L-lactate on fAHP (**j**), mAHP (**k**) and sAHP (**l**) by IbTx pretreatment. Left, representative firing traces. Scale bars: 10 s, 20 mV. *n* = 14 and 12 neurons from 5 mice per group. Student’s *t*-test, for fAHP, t_(24)_ = 0.903, *p* = 0.3755; for mAHP, t_(24)_ = 1.089, *p* = 0.287; for sAHP, t_(24)_ = 0.1261, *p* = 0.9007. **m** Diminished effects of L-lactate on neuronal excitability by IbTx pretreatment. Left, representative firing traces. Scale bars: 0.1 s, 20 mV. Right, quantitative data. *n* = 14 and 12 neurons from 5 mice per group. Two-way ANOVA, gene effect, F_(1168)_ = 0.6741, *p* = 0.4128. Data were shown as mean ± SEM. ***p < 0.001; ns, no significant difference. Source data are provided as a Source Data file.

We next sought to explore the underlying mechanisms by which L-lactate increases neuronal excitability and reduces depressive-like behaviors. Although we found that the firing frequencies were not altered with acute L-lactate exposure (Supplementary Fig. 11a, b), they were significantly elevated with longer incubation (>2 h) of L-lactate (Fig. 7a–c). In contrast, treatment with D-lactate, an isomer of L-lactate primarily synthesized in bacteria, showed little effect on neuronal excitability (Supplementary Fig. 11c–e). By analyzing the shape of action potentials, we found a significant decrease in the fast after-hyperpolarization (fAHP) with L-lactate treatment, leaving the amplitude, half-width, and threshold of action potentials unchanged (Fig. 7d–f). In contrast, the medium AHP (mAHP) and slow AHP (sAHP) were not altered (Fig. 7g–i). Consistent with these results, the fAHP amplitude was higher in Sus, *GFAP-Ldha*^*–/–*^, and GFAP-LDHA-KD mice than that in Res, *Ldha*^*f/f*^, and GFAP-YFP mice, respectively (Supplementary Fig. 11f–h). Together, these observations pinpoint that L-lactate regulates neuronal excitability via modulating fAHP.

It is well known that BK channels mediate fAHP^38^. Treatment with IbTx, a BK channel antagonist, significantly decreased fAHP levels, and increased the neuronal firing frequencies with no change in membrane potential, input resistance, and levels of mAHP and sAHP (Supplementary Fig. 12). Noticeably, L-lactate treatment failed to alter the fAHP, firing frequencies as well as mAHP and sAHP in the IbTx-treated group (Fig. 7j–m). These data suggest that the effects of L-lactate on neuronal firing depend on BK channels.

### BK channel in the dmPFC mediates the effects of L-lactate on depressive-like behaviors

**Fig. 8:**
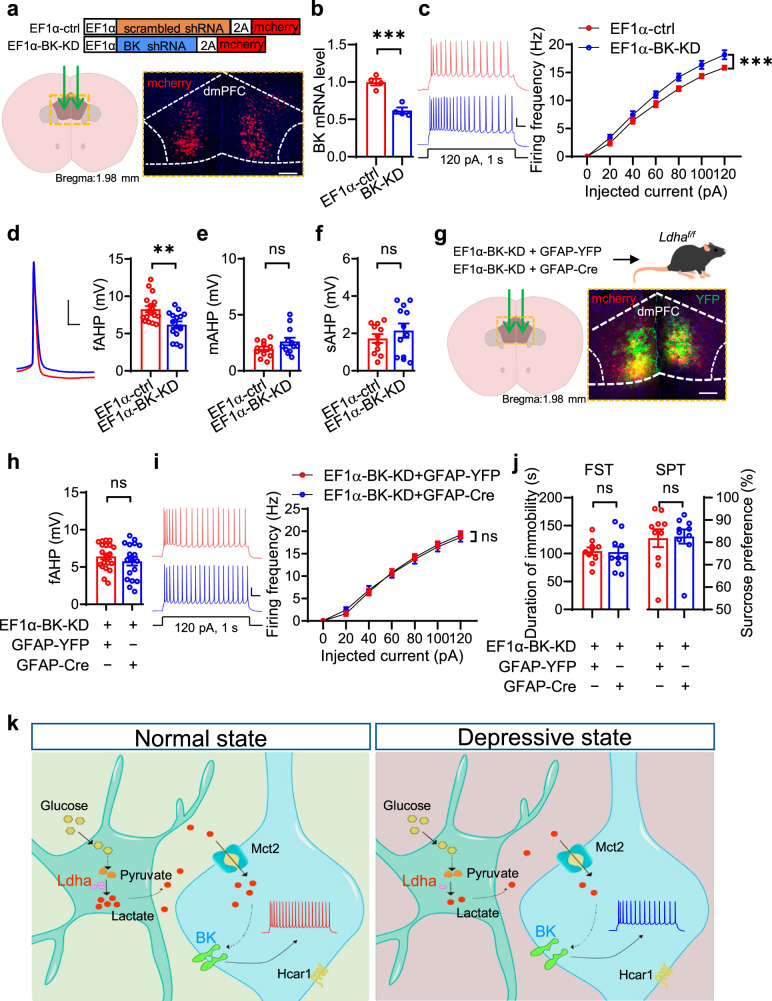
BK channels in the dmPFC are required for L-lactate-mediated regulation of depressive-like behaviors. **a** Illustration of viral injections. Scale bar, 100 µm. **b** Reduced *BK* mRNA level. *n* = 4 mice per group. Student’s *t*-test, t_(6)_ = 6.309, *p* = 0.0007. **c** Increased neuronal excitability in EF1α-BK-KD mice. Left, representative firing traces. Scale bars: 0.1 s, 20 mV. Right, quantitative data. *n* = 17 and 16 neurons from 4 EF1α-ctrl and 4 EF1α-BK-KD mice. Two-way ANOVA, gene effect, F_(1,217)_ =  26.5, *p* = 0.0000. **d** Reduced fAHP in EF1α-BK-KD mice. Left, representative firing traces. Scale bars: 10 ms, 20 mV. Right, quantitative data. *n* = 17 and 16 neurons from 4 EF1α-ctrl and 4 EF1α-BK-KD mice. Student’s *t*-test, t_(31)_ = 3.409, *p* = 0.0018. **e**, **f** Not changed mAHP (**e**) or sAHP (**f**) in EF1α-BK-KD mice. *n* = 11 and 12 neurons from 4 EF1α-ctrl and 4 EF1α-BK-KD mice. Student’s *t*-test, for mAHP, t_(21)_ = 1.606, *p* = 0.1232; for sAHP, t_(21)_ = 0.9175, *p* = 0.3693. **g** Illustration of viral injections. Scale bar, 100 µm. **h** Diminished effect of LDHA deficiency on fAHP by knockdown of BK. *n* = 20 and 18 neurons from 5 EF1α-BK-KD + GFAP-YFP and 5 EF1α-BK-KD + GFAP-Cre mice, respectively. Student’s *t*-test, t_(36)_ =  0.9864, *p* = 0.3305. **i** Diminished effect of LDHA deficiency on neuronal excitability by knockdown of BK. Left, representative firing traces. Scale bars: 0.1 s, 20 mV. Right, quantitative data. *n* = 23 and 20 neurons from 5 EF1α-BK-KD + GFAP-YFP and 5 EF1α-BK-KD + GFAP-Cre mice, respectively. Two-way ANOVA, gene effect, F_(1,287)_ = 0.0204, *p* = 0.8866. **j** Diminished effect of LDHA deficiency by knockdown of BK on the immobility duration and sucrose preference. *n* = 10 mice per group. Student’s *t*-test, for FST, t_(18)_ = 0.1399, *p* = 0.8903; for SPT, t_(18)_ = 0.1502, *p* = 0.8823. **k** Proposed working model. Data were shown as mean ± SEM. **p < 0.01, ***p < 0.001; ns, no significant difference. Source data are provided as a Source Data file.

To further test the involvement of BK channels, we generated AAVs that express BK-shRNA targeting the transcript of BK α subunits^39^ (Fig. 8a). The *BK* mRNA level was reduced in the dmPFC of mice injected with BK-shRNA virus (Refer to as EF1α-BK-KD mice) (Fig. 8b). In addition, EF1α-BK-KD mice exhibited elevated firing frequencies, decreased fAHP and similar mAHP and sAHP in comparison to those mice injected with scrambled shRNA virus (Refer to as EF1α-ctrl mice) (Fig. 8c–f). These data prove the importance of BK channels in regulating neuronal excitability.

To determine whether knockdown of BK could diminish the effects of LDHA deficiency on neuronal activity and depressive-like behaviors, we injected GFAP-Cre and BK-shRNA viruses simultaneously into the dmPFC of *Ldha*^*f/f*^ mice (Fig. 8g). LDHA deficiency failed to alter fAHP, neuronal activity, and depressive-like behaviors when BK was knocked down in the dmPFC (Fig. 8h–j). Altogether, these findings demonstrate that BK channels mediate the effects of L-lactate on neuronal excitability and depressive-like behaviors.

## Discussion

Here we discover that astrocytic LDHA plays an important role in regulating neuronal excitability and depressive-like behaviors via shuttling lactate. Besides, we identify a downstream target mediating the anti-depressant effects of L-lactate. In our working model, social-defeat stress down-regulates LDHA expression in the dmPFC; LDHA deficiency in astrocytes reduces L-lactate production, leading to impaired neuronal excitability through elevation of BK channel-mediated fAHP, and eventually contributes to the development of depression-like phenotypes (Fig. 8k). Our study provides insights into the pathogenesis of depression and offers potential therapeutic targets.

Disturbed energy metabolism is widely reported in depression patients and animal models. Mounting evidence demonstrated impaired glucose metabolism in the mPFC of depression patients and animal models^11–13,40^. However, the role of glycolysis in the pathogenesis of depression has been largely ignored. In fact, a relatively high level of aerobic glycolysis happens in human PFC^19^, a critical region for depression^7^. Aerobic glycolysis in the brain declines during aging^41^, and has been implicated in several brain disorders, such as Alzheimer’s disease, Huntington’s disease, and stroke^42–44^. These observations suggest an important role of glycolysis in normal brain function. By using an unbiased proteomic analysis, genetic modification, and electrophysiological recordings, we demonstrate that reduced LDHA expression underlies the compromised glycolysis and decreased neuronal excitability under a depression state. Note that the reduced level of LDHA in the dmPFC of stress-susceptible mice is unlikely due to the cell loss as the numbers of neurons and astrocytes as well as the astrocytic volume were not different (Supplementary Fig. 13). Besides, moderate changes in levels of other glycolysis-related enzymes (such as En2) were also observed. The possible involvement of these enzymes in the pathogenesis of depression warrants future investigations. Moreover, it would be interesting to determine whether, except for its expression, the LDHA activity is also altered in response to social stress.

Astrocytes are the most abundant cells in the brain and play critical roles in neuronal development, synaptic plasticity, and maintenance of brain homeostasis^45^, whose dysfunctions are closely reported in various neuropsychiatric disorders, including depression^32,46^. Numerous studies have suggested that astrocytes release lactate, which acts as an energy substrate or a signaling molecule, to modulate neuronal functions^21^. Based on these results, the “lactate shuttle” theory has been proposed^20^. Notably, several studies have also reported that neuronal stimulation triggers neuronal glycolysis and does not rely on lactate originating from astrocytes^47,48^. While the exact reasons underlying such discrepancy are not clear, lactate shuttle from astrocytes to neurons might occur in a context-dependent manner and may differ between brain states, brain regions, and different developmental stages. In the present study, we observed reduced levels of extracellular lactate, neuronal excitability deficit, and depressive-like behaviors by *Ldha* deletion from astrocytes. Conversely, exogenous lactate infusion into the dmPFC significantly alleviates the depression phenotype. These data are in accord with previous reports showing the release of lactate from cortical astrocytes with antidepressant-fluoxetine treatment^49^, suggesting that disruption of lactate shuttle contributes to the pathogenesis of depression. A recent study has reported elevated lactate levels in the mPFC during the FST, suggesting a role of lactate in mediating coping responses to stress^50^. We speculate that lactate release may exist in time and/or intensity-dependent dynamics. As a compensatory mechanism, lactate levels up-regulate in response to acute stress to protect neurons from unrestrained damage. During overwhelming stress, disruption of lactate production and release occurs, which eventually aggravates the development of depression phenotypes. It is noteworthy that oligodendrocyte, another glial type in the brain, has also been reported to provide lactate to fuel neurons^23,51^, and dysfunctions of oligodendrocytes are implicated in depression pathogenesis^52^. It is plausible to speculate that astrocytes, may work in concert with oligodendrocytes, affect neuronal activities and depressive-like behaviors through lactate shuttle upon social stress. However, our evidence indicates little changes in *Ldha* mRNA and lactate levels in the oligodendrocytes from the three groups of CSDS mice (Supplementary Fig. 14), therefore against such speculation. Note that this view shall be taken with caution as the sample size is somewhat limited. Besides, we failed to measure LDHA protein levels in purified oligodendrocytes, most probably due to the restricted oligodendrocyte number in the gray matter. Nevertheless, the exact effects of social stress on oligodendrocytes, the functions of LDHA in oligodendrocytes, as well as oligodendrocytes-derived lactate, in the development of depressive-like behaviors await further careful investigation.

The underlying downstream mechanisms that execute the beneficial effects of lactate on depression are not clear. A recent study claimed lactate exerts anti-depressant effects via stimulating neurogenesis in the adult hippocampus^53^. While it offered a potential mechanism underlying the beneficial action of peripheral lactate on depression, it failed to provide the relationship between endogenous lactate homeostasis and depression. In our study, we report that lactate elevates neuronal excitability in the dmPFC via regulating BK channels. Through detailed analysis of action potential shape, we detected a reduction of fAHP levels by lactate treatment. Furthermore, using pharmacological and genetic approaches, we identified that BK channels mediate the effects of LDHA deficiency on neuronal excitability and the consequent depression phenotypes. Of note that a recent paper reports an acute promoting effect of lactate on the excitability of cortical neurons from juvenile rat^54^, which is in line with our findings. They further found that ATP-sensitive potassium (K_ATP_) channel was the main target for lactate signaling to regulate neuronal firing. Although we cannot exclude the possibility that the different results from ours may be attributed to the different brain regions (barrel cortex vs dmPFC) or ages (juvenile vs adult), an audacious speculation is that BK and K_ATP_ channels may work in concert with distinct kinetics (acute for K_ATP_ channel; chronic for BK channel as shown in the present study) to mediate lactate’s effects on neuronal firing, which are worth further investigation in the future study. Nevertheless, our results reveal a mechanism for lactate in the regulation of neuronal activity and depressive-like behaviors.

It remains to be determined what mechanisms are involved in the regulation of BK channels by lactate. A typical BK channel is formed by a tetramer of four pore-forming α subunits associated with auxiliary β subunits (β1–β4) and alteration in the composition of these subunits profoundly influences its function^55^. Notably, BK channels undergo fundamental epigenetic, post-transcriptional, and post-translational modifications including acetylation, pre-mRNA splicing, protein phosphorylation, and ubiquitination^56–58^. Noteworthy, a recent study has reported lactate as an epigenetic regulator modulating histones and thus affecting gene transcription^59^. It would be interesting to determine whether epigenetic mechanisms are involved in the regulation of BK channel function by lactate in future studies.

It is worth noting that a potential limitation of the present study is that the functions of astrocytic LDHA for depressive-like behaviors were examined in stress naive rather than stressed mice, as previous research has indicated that the antidepressant treatments may exert different, sometimes even opposite effects in stressed vs unstressed animal^60,61^. Nevertheless, many previous studies have investigated the roles of important molecules of interest in depressive-like behaviors under stress-naive context^17,32,62^. These results suggest that malfunctions of critical genes/molecules in themselves could mediate depression symptoms. In the present study, we provide evidence to demonstrate a critical role of astrocytic LDHA in the regulation of neuronal activity and depressive-like behaviors through the lactate shuttle mechanism. Note that the rescue effect of overexpressing LDHA in stress-exposed mice could also support a causal role of LDHA. It will be interesting to test the higher susceptibility of LDHA-deficient mice in developing depression symptoms when being treated with sub-threshold stress^3,63^. Result as such may suggest additional function(s) of LDHA in the brain.

## Methods

### Animals

Animal experiments were carried out following the Guidelines for Animal Care and Use of China. The experimental animal protocol was approved by the Animal Ethics Committee of Guangzhou Medical University (Protocol number: GY2019-033). Eight to twelve-week-old male or female mice were used for experiments. C57BL/6J mice were purchased from Guangdong Medical Laboratory Animal Center. *Ldha-floxed* (*Ldha*^*f/f*^) (stock#: 030112) was purchased from Jackson Laboratory. *GFAP-Cre* and *Rosa-LSL-tdTomato* (*td*) mice were described previously^30,64^. The following primers were used for genotyping: *Ldha*^*f/f*^, 5′-CTG AGC ACA CCC ATG TGA GA-3′ and 5′-AGC AAC ACT CCA AGT CAG GA-3′; *GFAP-Cre*, 5′-ACT CCT TCA TAA AGC CCT-3′ and 5′-GCC AGC TAC GTT GCT CAC TA-3′. All mice were housed under a standard 12 h light/dark cycle (light on at 6:00; off at 18:00) with a temperature of 22 ± 1 °C and >30% humidity and received food (Protein, 22.47%; fat, 12.11%; Carbohydrate, 65.42%) and water *ad libitum*.

### Reagents and antibodies

Chemicals were purchased from Sigma-Aldrich unless otherwise indicated. 6-cyano-7-nitroquinoxaline-2,3-dione (CNQX, 0190), DL-2-amino-5-phosphonopentanoic acid (DL-AP5, 0105), and SR-95531 (1262) were purchased from Tocris Bioscience. The following primary antibodies were used: rabbit polyclonal anti-LDHA (Cell Signaling Technology, 2012S; 1:1000 for blotting); mouse monoclonal anti-LDHB (Santa Cruz Biotechnology, sc-100775, 431.1; 1:1000 for blotting); mouse monoclonal anti-α-Tubulin (Santa Cruz Biotechnology, sc-5286, B-7; 1:1000 for blotting); mouse monoclonal anti-GFAP (Millipore, MAB360, GA5; 1:1000 for blotting); mouse monoclonal anti-Tuj1 (Cell Signaling Technology, 4466, TU-20; 1:1000 for blotting); mouse monoclonal anti-NeuN (Millipore, MAB377, A60; 1:200 for immunofluorescent staining); rabbit monoclonal anti-S100ß (abcam, ab52642, EP1576Y; 1:200 for immunofluorescent staining).

### CSDS model

CSDS mouse model was utilized as described previously^65^. In brief, experimental mice were single-housed for one week before CSDS training. During training, each mouse was exposed to a daily-changed aggressive CD1 mouse for 5–10 min for ten consecutive days. Following the daily defeat, the mouse remained in the CD1 mouse’s homecage, except it was separated from the aggressor by a perforated translucent plastic divider. The control mice were housed in pairs by the same divider without exposure to CD1 mice and relocated to a cage with a novel control mouse each day. Social interaction test was performed 24 h after the last social defeat.

### Behavioral tests

The social interaction test (SIT) consists of two phases, with each lasting for 150 s. During the first phase, the experimental mice were allowed to freely explore the arena [42 cm (w) × 42 cm (d) × 42 cm (h)] containing an empty wire-mesh enclosure [10 cm (w) × 6.5 cm (d) × 42 cm (h)], which was located right above the center of the arena (no target). Mice were returned to their homecages for 30 s. During the second phase, they were re-introduced to the arena with an unfamiliar aggressive CD-1 mouse placed in the enclosure (target). The interaction zone (IZ) is defined as a 14 cm × 24 cm rectangular arena that projects 8 cm around the enclosure. The mice were video-monitored using Ethovision XT 14.0 software (Noldus). Sus and Res mice were separated by the social interaction ratio (SIR), which is defined as (interaction time, target) / (interaction time, no target). Mice with SIR > 1 were considered as Res; those with SIR < 1 were considered as Sus.

In the FST, mice were placed in a transparent cylinder [12 cm (d) × 25 cm (h)], filled with water (temperature 25 ± 1 °C) to a depth of 15 cm. Mice were monitored using Ethovision XT 14.0 software for 6 min and the duration of immobility during the last 4 min was recorded.

The SPT was performed according to a previous report^66^. Briefly, mice were individually housed with ad libitum food for the two-day test period. During the test, mice were exposed to one bottle with 2% sucrose and the other with water for 24 h, followed by switching the bottle position. Every 24 h, the amounts of sucrose and water consumed were recorded. The consumption of each fluid was assessed, and sucrose preference (%) was calculated as sucrose consumption/(sucrose + water consumption) × 100%.

In the open field test, mice were placed in an arena (40 × 40 × 20 cm), and their movement was monitored for 30 min using an overhead camera and tracking software (Ethovision XT 14.0). The central region (20 × 20 cm) of the arena was defined as the center. The number of entries and duration spent in the central region was noted.

An Elevated plus-maze, consisting of two opposing wall‐closed arms and two open arms at 5 × 30 cm, was placed ~50 cm above the floor. During the test, each mouse was gently placed in the central area with its nose facing one of the closed arms. The time that mice spent in the open arm, closed arm, and center area, and the number of entries was quantified autonomously using Ethovision XT 14.0 software.

The T-maze barrier choice task was performed according to previous reports with minor modification^67^^,^^68^. In brief, all mice were food-restricted to 80–85% of their baseline weight throughout the experiments to motivate learning. The T-maze consists of three arms (30 × 10 × 20 cm). On day 1, mice were allowed to freely explore the T-maze for 5 min for habituation. During the training phase 1 (day 2), a food pellet (20 mg) was placed in the right arm, and mice were placed in the start arm and trained to access the pellet for 20 times. A success was defined as mice turning into the right arm immediately after being placed in the start arm. On the third day, a low plastic barrier (triangle, 4.5 cm high) covered with nylon wire was placed in the right arm, 5 cm away from the gate of the right arm. Mice were allowed to climb over the barrier to achieve the food pellet. An assistance was needed in case mice failed to climb over the barrier during the first trial. Mice were trained for 20 times. A success was defined as mice climbing over the barrier in 5 s after arriving the barrier. On the test day (day 4), a high barrier (8.5 cm high) was utilized to replace the low one. All the procedures were the same as on day 3. The success rate as calculated as the success number / total trial number.

The social interaction was detected using the three-compartment social interaction test as described previously^69^. Briefly, a rectangular Plexiglas arena (50 × 25 × 25 cm) was divided into three compartments (left and right compartments: 20 × 25 cm). Two enclosures that allow for visual, auditory, olfactory and tactile contact were placed in the top left and top right corners of the left and right compartments, respectively. In the pre-test, two enclosures were empty, and the experimental mouse was allowed to freely explore the arena for 5 min. On the second day, a C57 male mouse (social stimulus) of similar age was placed in the right enclosure with the left one kept empty. The experimental mouse was put back into the arena for free exploration for 5 min. All the sessions were video-tracked using Ethovision XT 14.0 (Noldus). The time that mice spent in each compartment was recorded, and the social interaction ratio was calculated as time in the social compartment / time in the empty compartment.

### Corticosterone measurement

Adult male mice were anesthetized with isoflurane, eyeballs were removed, and trunk blood was collected into EP tubes and leaved at 4 °C overnight. After centrifugation at 420 g for 15 min at 4°C, serum supernatant was collected, and stored at -80°C until use. Corticosterone levels were assessed with an ELISA kit (JLC3617, Gelatins) according to the manufacturer’s instructions.

### Proteomic analysis

Proteomic analysis was performed as described in a previous report^70^. Briefly, dmPFC tissues were homogenized in a buffer that contains 8 M urea and 50 mM NH_4_HCO_3_ using an ultrasonic homogenizer (Thermo Fisher Scientific, Waltham). Samples containing 100 μg protein were incubated in the same buffer with 5 mM DTT, alkylated by 10 mM iodoacetamide, and digested in 2 μg trypsin (Promega, Madison, WI), at 37 °C overnight. Digested peptides were desalted using MonoSpin C18 desalination column (GL Sciences, Tokyo). 2 μg of each sample was analyzed on Q Exactive HF-X (Thermo Fisher Scientific, Waltham, MA).

Raw mass spectrum (MS) data were acquired and imported into MaxQuant version 1.5.4.1 to identify proteins and peptides and quantified using the stabilized large LFQ ratios with false discovery rate (FDR) < 0.01. The MS/MS spectra were run against the human UniProt FASTA database (release 2016_07, 49863 sequences) with trypsin/P set as the enzyme, which allowed two missed cleavage sites of trypsin. Mass errors were set to 5 ppm for precursor ions and 0.02 Da for-fragment ions. Carbamido-methylation on Cys was specified as the fixed modification with modification variables as oxidation (M), deamidation (NQ), acetylation (Protein N-term). The minimum peptide length was set at 7. The options of “Second peptides”, “Match between runs” and “Dependent peptides” were enabled. All other parameters were set as default values. Total peptide amounts on channels average were used for normalization and scaling.

A total of 3513 proteins were identified in the fractions at 1% FDR. Relative protein abundance ratios between groups were calculated using the average of normalized protein abundance values of the group and subjected to a two-tailed Student’s *t*-test (*p* < 0.05 was considered significant). The volcano plots and heatmap were prepared using the GraphPad 8 and Tbtools v1.09876 software^71^, respectively. We used Gene Ontology (GO) knowledgebase (http://geneontology.org) and ClusterProfiler to perform GO of biological processes and Kyoto Encyclopedia of Genes and Genomes (KEGG) protein enrichment analyses^72^. The enriched GO biological processes and KEGG pathway were identified and listed according to their enrichment *p*-value (*p* < 0.05). The mass spectrometry proteomics data have been deposited to the ProteomeXchange Consortium (http://proteomecentral.proteomexchange.org) via the iProX partner repository with the dataset identifier PXD037878.

### Glycolysis analysis

Glycolysis analysis was performed as mentioned in a previous report with minor modification^73^. Briefly, mice were anesthetized with isoflurane, and brain tissue was quickly collected and chilled in ice-cold modified artificial cerebrospinal fluid (ACSF, in mM) (140 NaCl, 3.5 KCl, 1.5 MgCl_2_, 2 CaCl_2_, 10 HEPES, and 10 glucose), whose pH value and osmotic pressure adjusted to 7.4 and 290–300, respectively. Coronal brain slices comprising dmPFC (200 µm, 2–3 slices) were sectioned using a VT-1000S vibratome (Leica, Germany), and recovered at a storage chamber containing the same ACSF at 32 °C for 30 min. The tissue was then micro-dissected by microforceps and transferred to an incubation chamber containing the same ACSF except for the absence of glucose at room temperature (24 ± 1 °C) for an additional 30 min before glycolytic tests. All solutions were continuously bubbled with 100% O_2_ (vol).

Extracellular acidification rate (ECAR) was measured using an XFe24 analyzer (Agilent) and Seahorse XF glycolysis stress kit (Agilent, #103020-100). Glucose (100 mM), oligomycin (10 µM) and 2-deoxy-D-glucose (2-DG, 50 mM) were freshly prepared using O_2_-saturated ACSF without glucose before use and preloaded with volumes of 75 µl, 83 µl and 93 µl into reagent delivery chambers A, B and C, respectively. dmPFC tissue was individually inserted into 20 wells of 24-well XF Islet Capture Microplates (Agilent, #103518-100) containing 675 µl of O_2_-saturated ACSF without glucose and covered by the nylon insert which was pre-incubated in the same ACSF, leaving the rest 4 wells served as blank controls with no slices. The microplate containing dmPFC was then loaded into the XFe24 analyzer and equilibrated for 15 min by three cycles including 3 min mix and 2 min wait before the first measurement. A measurement cycle consisted of 3 min mix, 2 min wait and 3 min measurement. After 24 min of baseline measurement, glucose, oligomycin and 2-DG were sequentially injected into the well at intervals of 24 min. The level of glycolysis was calculated as the last ECAR after glucose injection minus the baseline of ECAR before glucose injection. The glycolytic capacity was calculated as the last ECAR after oligomycin injection minus the baseline of ECAR. Each value was normalized by the protein concentration of corresponding sample.

### Extracellular lactate measurement

The lactate concentration was determined by an enzymatic reaction which results in a colorimetric (570 nm) product when lactate is converted to pyruvate using the lactate assay kit (Sigma, #MAK064). Briefly, dmPFC (~5 mg) was acutely micro-dissected after mice were anesthetized and incubated in the 1.5 ml-EP tubes with 80 µl of ice-cold oxygenized ACSF (see formula above) for 12 min. The ACSF was then collected and subjected to lactate measurement according to the manufacturer’s instructions. All experiments were carried out in duplicate. Absorbance was measured at 570 nm using a microplate reader (TECAN, Infinite 200 PRO). The lactate level, determined from the standard curve which was obtained from standard lactate samples (0, 2, 4, 6, 8, 10 mM), was normalized by the protein amount of corresponding sample.

### Western blot

Tissues were micro-dissected and homogenized in a RIPA Buffer containing (in mM): 50 Tris-HCl, pH 7.4, 150 NaCl, 2 EDTA, 1 PMSF, 50 sodium fluoride, 1 sodium vanadate, 1 DTT with 1% sodium deoxycholate, 1% SDS and 1% protease inhibitors cocktails.

Each sample with protein level at 20 μg was then resolved using 12% of SDS/PAGE and transferred to nitrocellulose membranes, incubated in TBS buffer containing 0.1% Tween-20 and 5% milk for 1 h at room temperature before incubation with primary antibodies (overnight at 4 °C). After washing, the membranes were incubated with Goat polyclonal HRP-conjugated secondary antibodies (Beyotime, anti-rabbit, A0208, 1:1000; anti-mouse, A0216, 1:1000) in the same TBS buffer for 1 h at room temperature. Immunoreactive complex bands were visualized using enhanced chemiluminescence (Pierce) and captured using the Genesys imaging system (Gene Company Limited). To quantify the band densities, the gray background values were subtracted from the gray values of proteins of interest using image J 1.53 g and then normalized with their loading controls. α-Tubulin was used as a loading control. All original blots were present in Source data file.

### Brain morphological analysis

Anesthetized mice were transcardially perfused with PBS followed by 4% PFA, brains were removed and fixed in 4% PFA at 4 °C for at least 8 h. Brain blocks were dehydrated in 30% sucrose solution, frozen in OCT at −80 °C freezer, and then sectioned into 30-μm-thick sections using a cryostat (CM1950, Leica). After permeabilization with 0.3% Triton X-100 and 5% BSA in PBS, sections were incubated with primary antibodies at 4 °C overnight. Following washing with PBS thrice, samples were incubated with donkey Alexa Fluor-conjugated polyclonal secondary antibodies (Jackson ImmunoResearch; anti-rabbit, 711-586-152, 1:500; anti-mouse, 715-545-150, 1:500) for 1 h at room temperature. Samples were mounted with Vectashield mounting medium (Vector lab) and images were captured using Nikon A1 confocal microscope. For analysis of astrocyte volume, z-stack images of S100β^+^ cells were acquired with 60x water immersion lens at step size of 0.25 µM. The cell surface was reconstructed using Imaris 9.0.1 software to estimate the astrocyte volume.

### Electrophysiological recording

As reported previously^30^, adult male mice were anesthetized with isoflurane, and brains were quickly removed and chilled in ice-cold modified artificial cerebrospinal fluid (ACSF) that contains (in mM): 120 Choline-Cl, 2.5 KCl, 7 MgCl_2_, 0.5 CaCl_2_, 1.25 NaH_2_PO_4_, 25 NaHCO_3_, and 10 glucose. Coronal slices (300 µm) containing dmPFC were sectioned in the same ice-cold modified ACSF using a VT-1000S vibratome (Leica, Germany) and transferred to an incubation chamber containing regular ACSF (in mM) (126 NaCl, 3 KCl, 1 MgSO_4_, 2 CaCl_2_, 1.25 NaH_2_PO_4_, 26 NaHCO_3_, and 10 glucose) at 32 °C for 30 min. The chamber containing slices stayed at room temperature (24 ± 1 °C) for an additional 1 h before recording. All solutions were saturated with 95% O_2_ / 5% CO_2_ (vol/vol).

During recording, slices were transferred to a recording chamber that was continuously superfused (2 ml/min) with ACSF. Whole-cell patch-clamp recording from pyramidal neurons in 2–3 layers of dmPFC was visualized with infrared optics using an upright microscope equipped with an infrared-sensitive CCD camera (DAGE-MTI, IR-1000E). Pipettes were pulled by a micropipette puller (P-97, Sutter instrument) with a resistance of 3–5 MΩ. Recordings were made with MultiClamp 700B amplifier and 1440A digitizer (Molecular Devices).

For mEPSC recording, pyramidal neurons were held at −70 mV in the presence of 20 μM RS-95531 and 1 μm TTX, with the pipette solution containing (in mM): 125 Cs-methanesulfonate, 5 CsCl, 10 Hepes, 0.2 EGTA, 1 MgCl_2_, 4 Mg-ATP, 0.3 Na-GTP, 10 phosphocreatine and 5 QX314 (pH 7.40, 285 mOsm).

To record mIPSC, pyramidal neurons were held at -70 mV in the presence of 20 μM CNQX, 100 μM AP-5 and 1 μm TTX, with the pipette solution containing (in mM): 140 CsCl, 10 Hepes, 0.2 EGTA, 1 MgCl_2_, 4 Mg-ATP, 0.3 Na-GTP, 10 phosphocreatine and 5 QX314 (pH 7.40, 285 mOsm).

The excitability of dmPFC pyramidal neurons was detected in current-clamp mode and measured by injecting a series of depolarizing pulses (from 0 pA to 120 pA, at a step of 20 pA), with the pipette solution containing (in mM): 125 K-gluconate, 5 KCl, 10 HEPES, 0.2 EGTA, 1 MgCl_2_, 4 Mg-ATP, 0.3 Na-GTP and 10 phosphocreatine (pH 7.40, 285 mOsm). The spike threshold, amplitude, and fAHP were calculated using the second current-evoked spike in response to 60-pA current injection since the first action potential was usually distorted because of the passive Vm rise caused by current injection, as reported previously^74^. The fAHP was defined as: the peak of the spike−the threshold. The medium and slow AHP (mAHP/sAHP) were recorded by injecting a current pulse (200 ms, 400 pA). The mAHP was defined as: the peak of AHP following the current pulse−baseline membrane potential. The sAHP was defined as: the membrane potential of an averaged time window from 280 to 320 ms after the current pulse−baseline membrane potential. Membrane input resistance was calculated in response to a series of hyperpolarizing pulses.

In the experiment of acute lactate treatment, after 10 min of baseline recording of firings, the superfused ACSF was switched to that containing (in mM): 116 NaCl, 10 Na-lactate, 3 KCl, 1 MgSO_4_, 2 CaCl_2_, 1.25 NaH_2_PO_4_, 26 NaHCO_3_, and 10 glucose, followed by firing recording for 30 min. In the experiment of chronic lactate or D-lactate treatment, brain slices were subjected to recordings after incubation for 2 h in the ACSF containing lactate as described above. In the control (ctrl) group, brain slices were incubated in the regular ACSF for 2 h and then subjected to recording.

In all experiments, series resistance was maintained below 20 MΩ and not compensated. Cells would be rejected if membrane potentials were positive more than −60 mV; or if series resistance fluctuated more than 20% of initial values. Data were filtered at 2 kHz and sampled at 10 kHz.

### Neuron and astrocyte culture

Neurons and astrocytes were cultured as described previously^30^. Briefly, mouse cortices were dissected from E18 pups and digested in 0.25% trypsin at 37 °C for 30 min. Dissociated cells were resuspended in the media (DMEM/F-12 50:50 supplemented with N2 and 10% FBS) and plated onto poly-lysine–coated 10-cm culture dishes for 4 h before replacing the medium with maintenance medium (neural basal medium supplemented with B27) and cytosine arabinoside (10 μM) to inhibit astrocyte proliferation. 50% of the medium was changed every 4–5 d. On DIV 12, the neuronal lysates were collected and subject to western blotting experiment.

For astrocytes cultures, cortices were dissected from P2–3 pups and digested in 0.25% trypsin at 37 °C for 30 min. Dissociated cells were resuspended in the media (DMEM and 10% FBS) and plated into a culture flask for 7–9 d. The flask was shaken at 250 rpm for 2 h to remove microglial cells and shaken for overnight to remove oligodendrocytes. Astrocytic lysates were then collected for western blotting experiment.

### Stereotactic surgery, cannula implantation, and virus injection

Adult male mice were anesthetized with isoflurane (2–3%) and head-fixed in a stereotaxic device (RWD Life Science. Inc). An incision was made in the scalp and two small holes, one on each side, were drilled into the skull. For cannula implantation, the guide cannula (ID: 0.38 mm; RWD Life Science) together with a dummy cannula was unilaterally implanted inside the right dmPFC (coordinates from bregma: AP, +1.98 mm; DV, –2 mm; ML, –0.3 mm) and cemented onto the skull with dental cement. Mice were then returned to their homecages for at least one week. On each injection day, 1 μl of sodium-lactate, freshly prepared in O_2_-saturated ACSF at a concentration of 10 mM, was injected through an internal cannula (ID: 0.2 mm, RWD Life Science) controlled by a microinjector (Nanoliter 2010, World Precision Instruments) at a slow rate of 0.2 μl/min. The capillary was slowly retracted 10 min after injection.

For virus injection, a specific volume of the virus was injected bilaterally into the dmPFC (coordinates from bregma: AP, +1.98 mm; DV, –2 mm; ML, ±0.3 mm) through a pulled glass capillary, controlled by the same microinjector at a slow rate of 10 nl/min. The capillary was slowly retracted 10 min after injection. Mice were returned to their homecages to recover from anesthesia under a heating pad and subjected to subsequent experiments after at least 3 weeks.

The recombinant adeno-associated viral (AAV) vectors were constructed and packed by Shanghai Sunbio Medical Biotechnology (Shanghai, China), unless otherwise indicated. Used vectors in the present study contain: EF1α-LDHA-KD (AAV2/9-EF1α::LDHA-shRNA-eGFP, titre: 3.09 × 10^12 ^v.g./ml, dilution: 1:2, 0.1 μl bilateral into dmPFC) and EF1α-ctrl (AAV2/9-EF1α::scrambled shRNA-eGFP, titre: 1.73 × 10^12 ^v.g./ml, dilution: 1:1, 0.1 μl bilateral into dmPFC); LDHA-OE (AAV2/9-CMV::LDHA-eGFP, titre: 4.95 × 10^12 ^v.g./ml, dilution: 1:4, 0.1 μl bilateral into dmPFC) and eGFP (AAV2/9-CMV::eGFP, titre: 2.12 × 10^12^ v.g./ml, dilution: 1:1, 0.1 μl bilateral into dmPFC); CaMKIIα-LDHA-KD (AAV2/9-CaMKIIα::LDHA-shRNA-eGFP, titre: 5.49 × 10^13^ v.g./ml, dilution: 1:10, 0.1 μl bilateral into dmPFC) and CaMKIIα-ctrl (AAV2/9-CaMKIIα::scrambled shRNA-eGFP, titre: 3.85 × 10^13 ^v.g./ml, dilution: 1:10, 0.1 μl bilateral into dmPFC); GFAP-Cre (AAV2/9-GFAP::Cre, titre: 2.45 × 10^12 ^v.g./ml, dilution: 1:400, 0.1 μl bilateral into dmPFC, BrainVTA) and GFAP-YFP (AAV2/9-GFAP::YFP, titre: 5.74 × 10^12 ^v.g./ml, dilution: 1:200, 0.1 μl bilateral into dmPFC, BrainVTA); CAG-HCAR1-KD (AAV2/9-CAG::HCAR1-shRNA-eGFP, titre: 1.12 × 10^13^ v.g./ml, 0.1 μl bilateral into dmPFC) and CAG-ctrl (AAV2/9-CAG::scrambled shRNA-eGFP, titre: 1.73 × 10^13 ^v.g./ml, 0.1 μl bilateral into dmPFC); CaMKIIα-MCT2-KD (AAV2/9-CaMKIIα::MCT2-shRNA-mcherry, titre: 4.32 × 10^13 ^v.g./ml, dilution: 1:3, 0.1 μl bilateral into dmPFC) and CaMKIIα-ctrl (AAV2/9-CaMKIIα::scrambled shRNA-mcherry, titre: 4.73 × 10^13 ^v.g./ml, dilution: 1:3, 0.1 μl bilateral into dmPFC); EF1α-BK-KD (AAV2/9-EF1α::BK-shRNA-mcherry, titre: 1.17 × 10^13 ^v.g./ml, dilution: 1:10, 0.1 μl bilateral into dmPFC) and EF1α-ctrl (AAV2/9-EF1α::scrambled shRNA-mcherry, titre: 3.99 × 10^13 ^v.g./ml, dilution: 1:20, 0.1 μl bilateral into dmPFC).

The shRNA target sequences of *Ldha*, *Hcar1*, *Mct2*, and *BK* have been reported in previous studies and are listed below: *Ldha*^75^, 5′-CCA GCA AAG ACT ACT GTG TAA-3′; *Hcar1*^76^, 5′-AAG ATG ACC AAA GTC CAG AGG-3′; *Mct2*^77^, 5′-TTC ATT GGA GGT TTA GGA TTA-3′; *BK*^39^, 5′-GCA CTT ACG TAC TGG GAA TGT-3′; *Scramble*, 5′-GTT CTC CGA ACG TGT CAC GTA-3′.

All viral vectors were aliquotted after receiving and stored at –80 °C until use.

### Quantitative RT-PCR analysis

Quantitative RT-PCR (qRT-PCR) was performed as described previously^65^. In brief, dmPFC tissue was acutely micro-dissected, and used for total RNA isolation using TRIzol reagent (15596-026, Invitrogen). RNA (1 µg) was reversely transcribed with oligo dT-primers using Maxima reverse transcriptase (EP 0742, Fermentas), followed by qPCR with SYBR Green detection (K 0222, Fermentas). Each sample was assayed in triplicates, and each plate contained loading standards in duplicate. The 2^−ΔΔCt^ method was utilized to analyze the mRNA levels. The mRNA levels of genes of interest were normalized to those of *GAPDH*. Sample size choice was based on previous studies^58,59^, not predetermined by a statistical method. Primer sequences were: *Hcar1*, 5′- GCA CGA TGT CAT GTT CCA GC -3′ and 5′- GGC TCC AAA CAA CGT TGA CC -3′; *Mct2*, 5′- AAT CTG GAG GCT GCT CTA CC -3′ and 5′- ATG TTT CTC TTG GCT GTT GTC AG -3′; *BK*, 5′- GGC TGG AAG TGA ATT CTG TAG-3′ and 5′- TGA GTA AGT AGA CAC ATT CCC -3′; *GAPDH*, 5′- GGT TGT CTC CTG CGA CTT CA -3′ and 5′- CCA CCA CCC TGT TGC TGT AG -3′.

### Oligodendrocyte isolation and flow cytometry

dmPFC tissues from 4 mice in each group were pooled together and digested into single cells with 1 mg/mL papain (HY-P1645, MCE) and 300 µg/mL DNase I (11284932001, Roche) for 30 min using program 37C_ABDK_01 of the gentleMACS Octo Dissociator with Heaters (Miltenyi Biotec) and sorted by magnetic beads with mouse monoclonal anti-O4 antibody (Miltenyi Biotec, 130-096-670, O4; 3 μl per 10^7^ total cells for sorting). To evaluate the purity of O4 positive cells, the sorted cells were incubated with mouse monoclonal anti-O4-PE antibody (R&D System, FAB1326P, O4; 10 μl per 10^6^ total cells for flow cytometry) and subjected to the flow cytometry assay using the CytoFLEX S.4 system (Beckman Coulter, USA).

### Statistics and reproducibility

Adult male mice (≥2 months), unless otherwise indicated, were used in the present study. Animal or replicate numbers for each experiment and results of the statistical analyses, including degrees of freedom and exact *p*-values are mentioned in the Figure legends. Experiments for western blot and brain morphological analysis were repeated independently with similar results for at least three times. Statistical analyses were performed using GraphPad Prism 8 (GraphPad Software). The sample size choice was made based on previous studies^30,64^. The student’s *t*-test and one-way ANOVA with Tukey’s post hoc test were used to compare data from two groups and more than two groups, respectively. Regular two-way ANOVA was used for electrophysiological studies that analyze more than two parameters. All tests were two-sided. All tests were two-sided, with a confidence level of 95%. Adjustments were made for multiple comparisons. All data represent mean ± SEM. A *p* < 0.05 was considered to be statistically significant.

### Reporting summary

Further information on research design is available in the Nature Portfolio Reporting Summary linked to this article.

## Supplementary information

Supplementary information

Reporting summary

## Data Availability

The raw mass spectrum data were run against the human UniProt FASTA database (release 2016_07, 49863 sequences). The proteomic data generated in this study have been deposited in the ProteomeXchange database with dataset identifier number of PXD037878. All data supporting the current study are provided in the Source data file. All additional information is available from the corresponding author upon request. Source data are provided with this paper.

## Source data

Source data
