# Supplementary material for: Astrocytic lactate dehydrogenase A regulates neuronal excitability and depressive-like behaviors through lactate homeostasis in mice
Source: Nat Commun. 2023 Feb 9;14:729. doi: 10.1038/s41467-023-36209-5 (PMC9911790; doi:10.1038/s41467-023-36209-5)
Supplement: Supplementary file 1 — Supplementary information [file 41467_2023_36209_MOESM1_ESM.pdf]

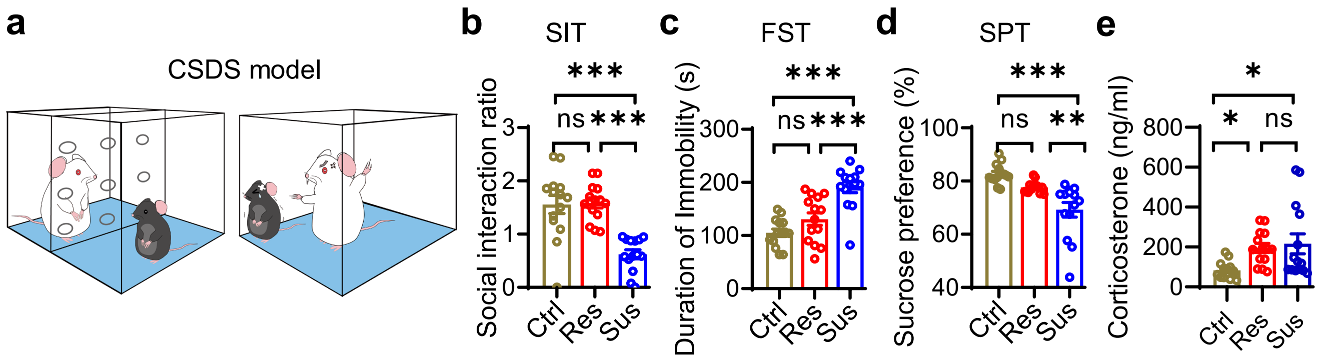

Supplementary Fig. 1. Characterization of depressive-like behaviors of CSDS model. **a** Diagram of the CSDS model. **b** Reduced social interaction ratio in Sus mice. n = 14 mice per group. One-way ANOVA followed by Tukey's post hoc test. $F_{(2,39)}$ = 20.05, p = 0.0000. Res vs Ctrl, p = 0.9821; Sus vs Res, p = 0.0000; Sus vs Ctrl, p = 0.0000. **c** Increased immobility duration in Sus mice in FST test. n = 14 mice per group. One-way ANOVA followed by Tukey's post hoc test. $F_{(2,39)}$ = 19. 6961, p = 0.0000. Res vs Ctrl, p = 0.173; Sus vs Res, p = 0.0003; Sus vs Ctrl, p = 0.0000. **d** Decreased sucrose preference in Sus mice in SPT test. n = 14 mice per group. One-way ANOVA followed by Tukey's post hoc test. $F_{(2,39)}$ = 16.12, p = 0.0000. Res vs Ctrl, p = 0.1029; Sus vs Res, p = 0.0031; Sus vs Ctrl, p = 0.0000. **e** Increased serum corticosterone level in both Res and Sus mice. n = 14 mice per group. One-way ANOVA followed by Tukey's post hoc test. $F_{(2,39)}$ = 4.866, p = 0.013. Res vs Ctrl, p = 0. 0499; Sus vs Res, p = 0.0162; Sus vs Ctrl, p = 0.887. Data were shown as mean ± SEM. *$p < 0.05$, **$p < 0.01$, ***$p < 0.001$; ns, no significant difference. Source data are provided as a Source Data file.

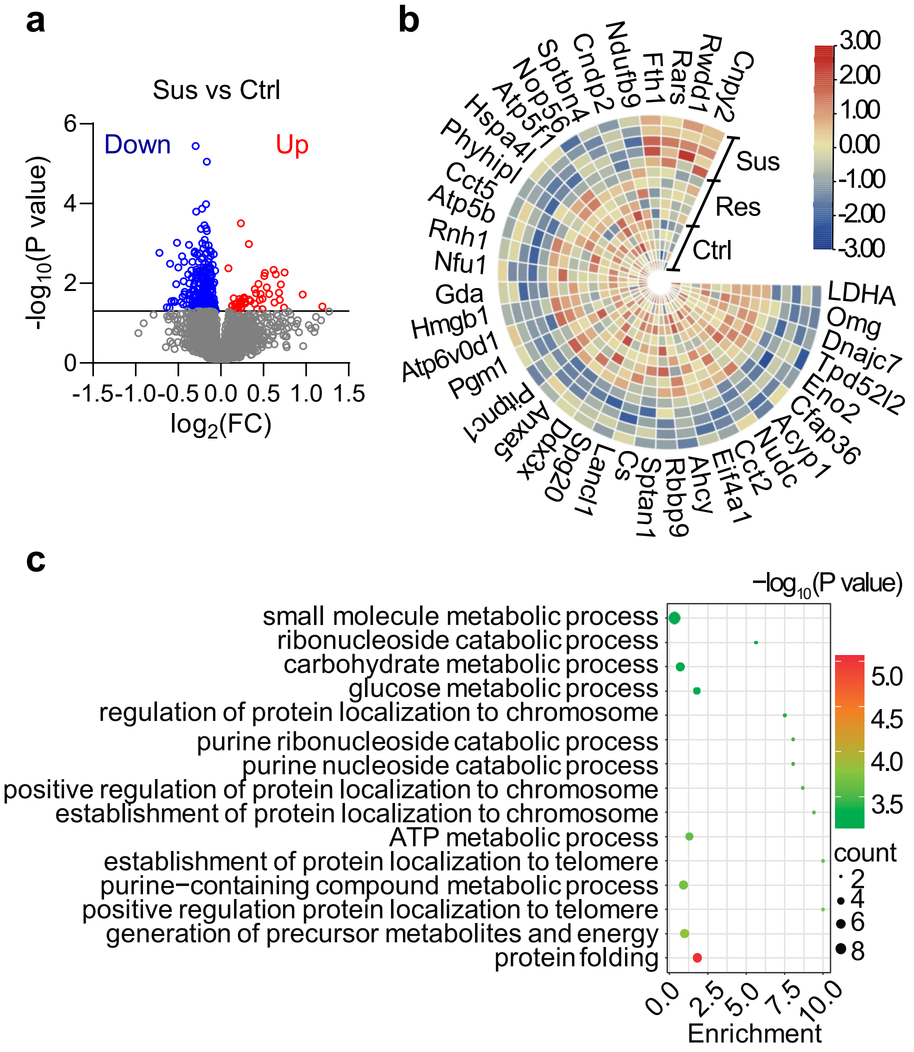

Supplementary Fig. 2. Gene ontology analysis of the common 38 DEPs. **a** Volcano plot of DEPs between Sus and Ctrl mice. Blue and red dots indicate significantly down- and upregulated proteins, respectively. **b** Heatmap of the 38 DEPs in Ctrl, Res and Sus groups. **c** Bubble plot of significantly enriched GO terms of the common 38 DEPs.

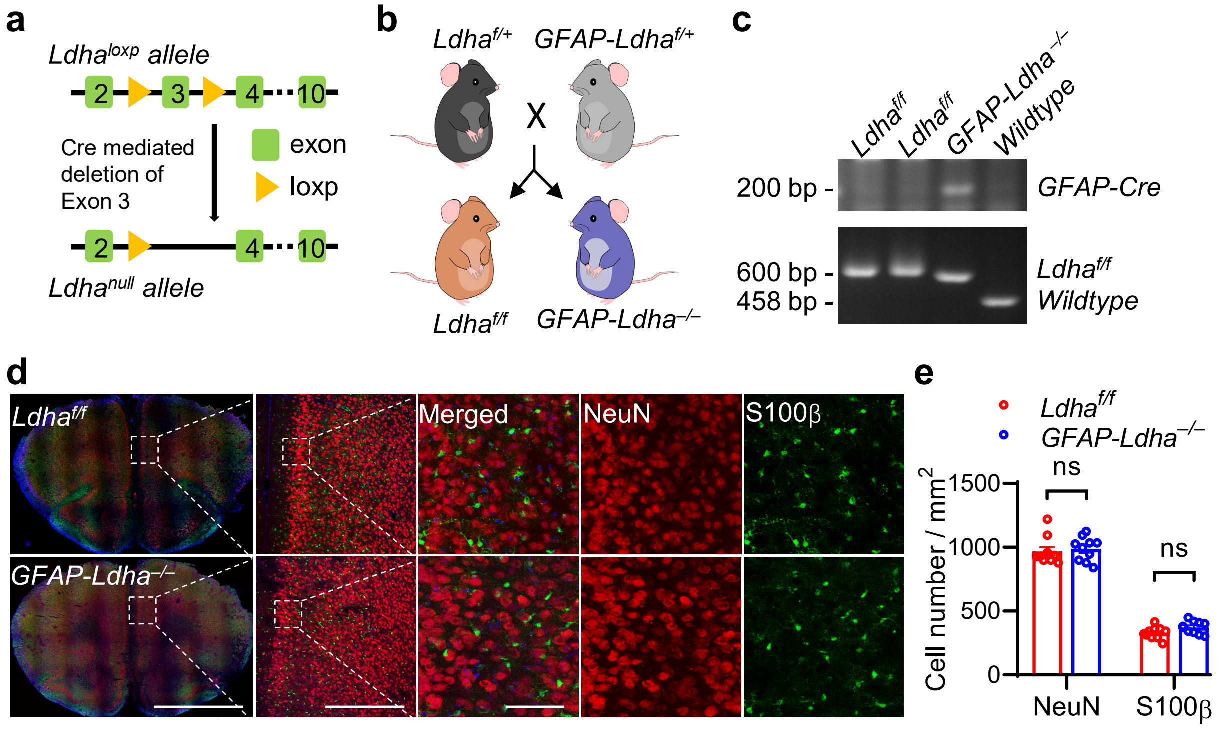

Supplementary Fig. 3. Characterization of *Ldha* brain mutant mice. a Diagram of conditional knockout strategy. The first panel is simplified genomic structure of *Ldha* gene. The second panel, targeted *Ldha* allele without exon 3 (*Ldha^{null}*). **b** Schematic diagram of breeding strategy to generate *Ldha* brain knockout (*GFAP-Ldha$^{-/-}$*) mice. **c** Genotyping results. Genomic DNA was collected from the mouse tail for PCR with indicated primers. *GFAP-Cre* primers generate a ~200 bp product. *Ldha* primers generate 600-bp products in *Ldha$^{f/f}$*, or 458-bp products in wildtype allele. **d** Representative immunostaining images. Brain slices containing dmPFC were co-stained with neuronal marker-NeuN (Red) and astrocytic marker-S100β (green), respectively. Dotted squares were enlarged and placed on the right. Scale bars: left, 1500 μm, middle, 200 μm, right, 50 μm. **e** Unaltered neuron and astrocyte numbers in *Ldha* brain mutant mice. *n* = 10 slices from 3 mice for each genotype. Student's t-test, for NeuN, $t_{(18)} = 0.4351$, $p = 0.6687$; for S100β, $t_{(18)} = 2.028$, $p = 0.0576$. Data were shown as mean ± SEM. ns, no significant difference. Source data are provided as a Source Data file.

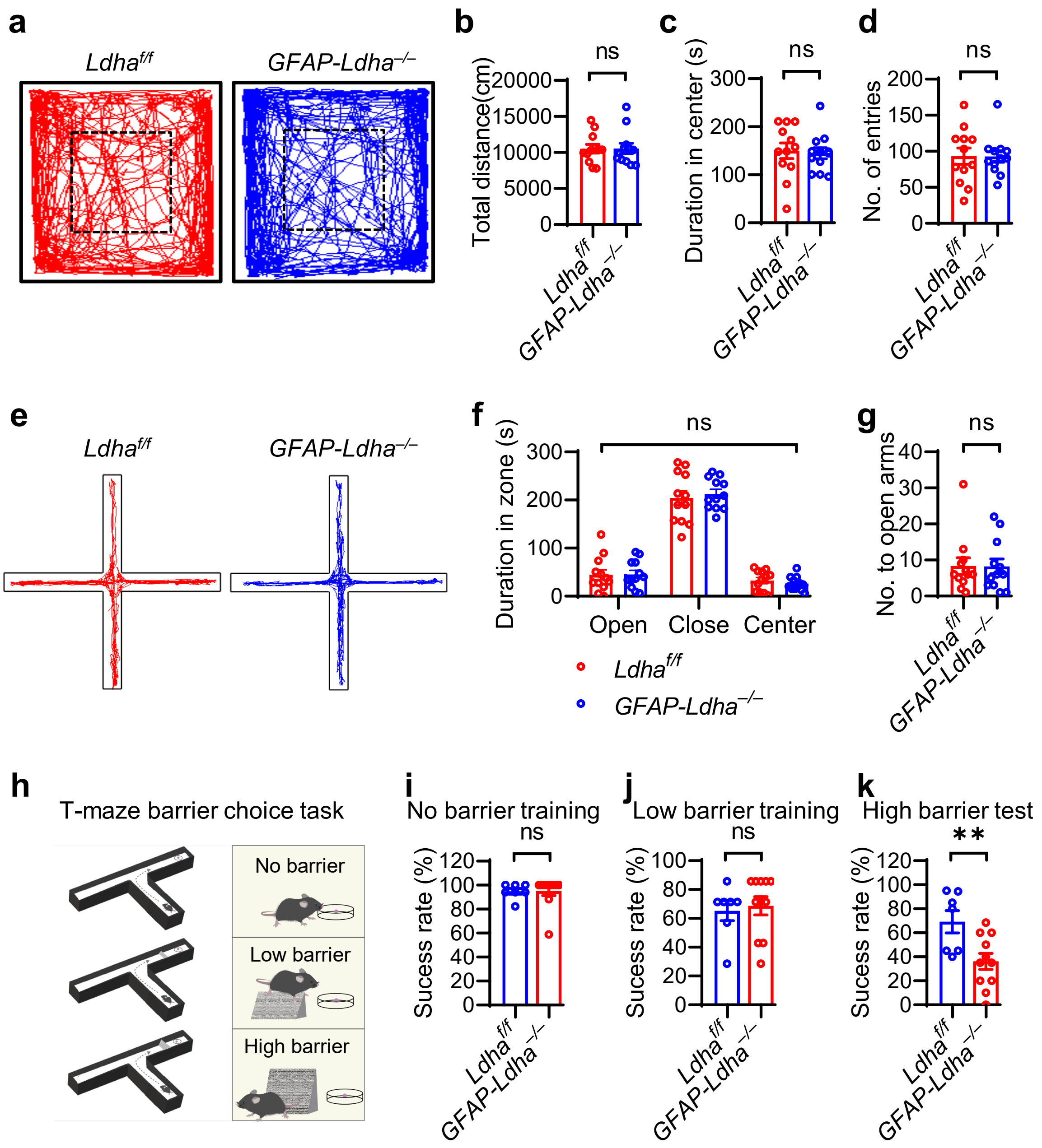

Supplementary Fig. 4. Effects of LDHA deletion on anxiety-like behaviors and reward function. a Representative trace that mice traveled in the open field test. **b** Similar distance that mice travelled in the open field test. *n* = 12 mice per genotype. Student's *t*-test, $t_{(22)}$ = 0.0513, *p* = 0.9596. **c** Unaltered duration in center. *n* = 12 mice per genotype. Student's *t*-test, $t_{(22)}$ = 0.2619, *p* = 0.7958. **d** Not changed number of entries to the center. *n* = 12 mice per genotype. Student's *t*-test, $t_{(22)}$ = 0.0304, *p* = 0.9761. **e** Representative traces that mice traveled in the elevated plus test. **f** comparable time spent in the open, close arms and center area. *n* = 12 mice per genotype. Two-way ANOVA, gene effect, $F_{(1,66)}$ = 0.013, *p* = 0.9

095. **g** Not changed frequency to open arm. *n* = 12 mice per genotype. Student's *t*-test, $t_{(22)}$ = 0.0273, *p* = 0.9785. **h** A diagram of T-maze barrier choice task. **i** Not changed success rate during training without barrier. *n* = 7 *Ldha*$^{f/f}$ mice and 11 *GFAP-Ldha*$^{-/-}$ mice. Student's *t*-test, $t_{(16)}$ = 0.0444, *p* = 0.9651. **j** Not changed success rate during training with a low barrier. *n* = 7 *Ldha*$^{f/f}$ mice and 11 *GFAP-Ldha*$^{-/-}$ mice. Student's *t*-test, $t_{(16)}$ = 0.3645, *p* = 0.7202. **k** Decreased success rate during test with a high barrier. *n* = 7 *Ldha*$^{f/f}$ mice and 11 *GFAP-Ldha*$^{-/-}$ mice. Student's *t*-test, $t_{(16)}$ = 2.944, *p* = 0.0095. Data were shown as mean ± SEM. ***p* < 0.01; ns, no significant difference. Source data are provided as a Source Data file.

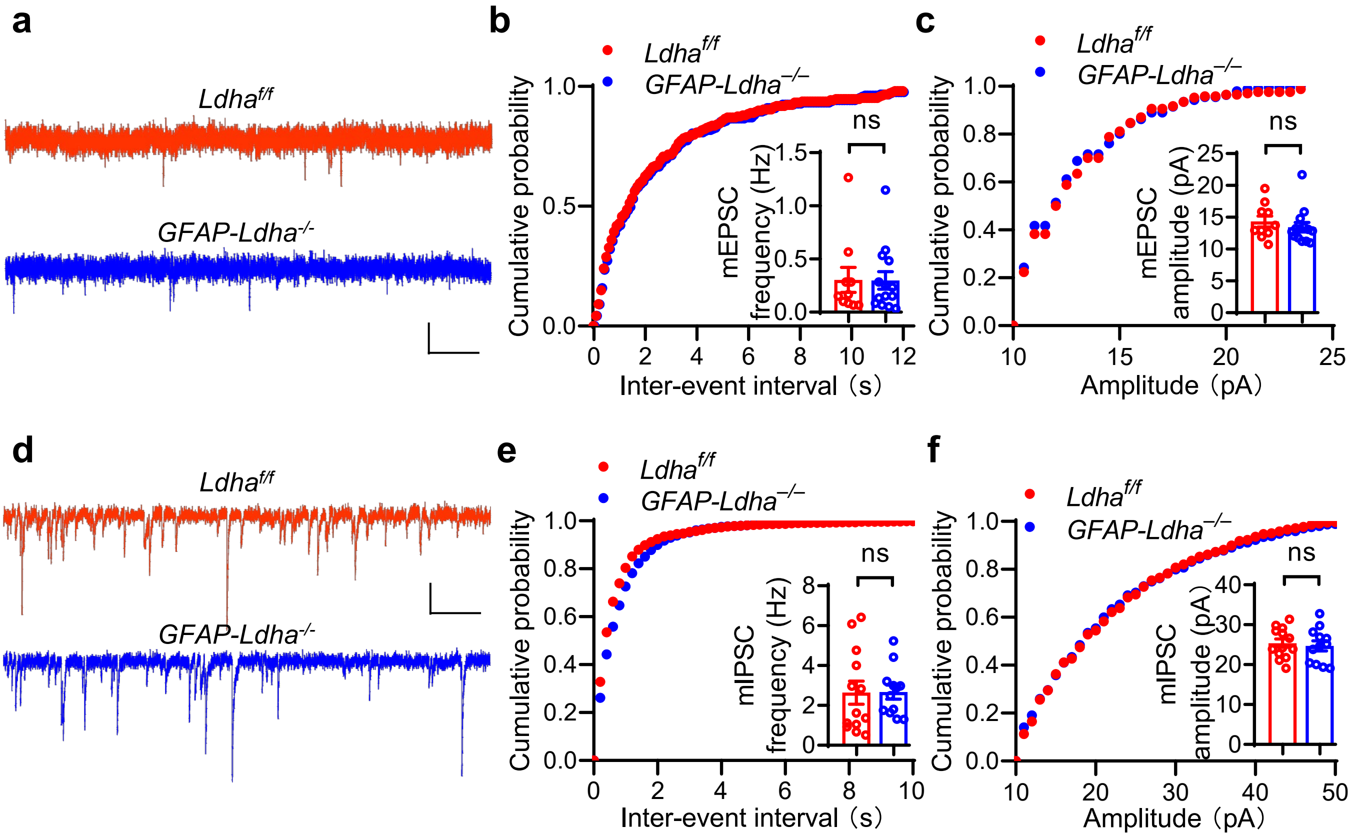

Supplementary Fig. 5. Limited effect of deletion of *Ldha* on synaptic transmission. a Representative miniature excitatory postsynaptic current (mEPSC) traces of dmPFC pyramidal neurons. Scale bars: 1 s, 10 pA. **b** Comparable mEPSC frequency between between *Ldha*$^{f/f}$ and *GFAP-Ldha*$^{–/–}$ mice. *n* = 10 and 14 neurons from 3 *Ldha*$^{f/f}$ and 3 *GFAP-Ldha*$^{–/–}$ mice, respectively. Student's *t*-test, $t_{(22)}$ = 0.0328, *p* = 0.9742. For the accumulative distribution analysis, K-S test, D = 0.09091, *p* = 0.6994. **c** Comparable mEPSC amplitude between *Ldha*$^{f/f}$ and *GFAP-Ldha*$^{–/–}$ mice. *n* = 10 and 14 neurons from 3 *Ldha*$^{f/f}$ and 3 *GFAP-Ldha*$^{–/–}$ mice, respectively. Student's *t*-test, $t_{(22)}$ = 0.7939, *p* = 0.4357. For the accumulative distribution analysis, K-S test, D = 0.2143, *p* = 0.5412. **d** Representative miniature inhibitory postsynaptic current (mIPSC) traces of dmPFC pyramidal neurons. Scale bars: 1 s, 10 pA. **e** Comparable mIPSC frequency between between *Ldha*$^{f/f}$ and *GFAP-Ldha*$^{–/–}$ mice. *n* = 13 and 12 neurons from 3 *Ldha*$^{f/f}$ and 3 *GFAP-Ldha*$^{–/–}$ mice, respectively. Student's *t*-test, $t_{(23)}$ = 0.0394, *p* = 0.969. For the accumulative distribution analysis, K-S test, D = 0.2745, *p*= 0.0429. **f** Comparable mIPSC amplitude between *Ldha*$^{f/f}$ and *GFAP-Ldha*$^{–/–}$ mice. *n* = 13 and

12 neurons from 3 *Ldha*$^{f/f}$ and 3 *GFAP-Ldha*$^{-/-}$ mice, respectively. Student's *t*-test, $t_{(22)}$ = 0.4096, p = 0.6859. For the accumulative distribution analysis, K-S test, D = 0.0732, p = 0.9999. Data were shown as mean ± SEM. ns, no significant difference. Source data are provided as a Source Data file.

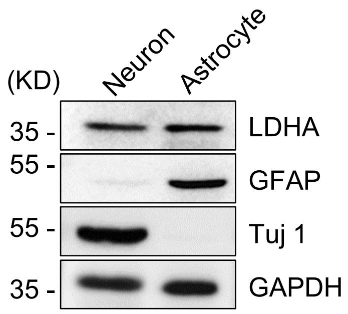

Supplementary Fig. 6. LDHA expression in different cell types in the brain. Western blotting results show that LDHA is expressed in both neurons and astrocytes. GFAP and Tuj1 serve as astrocyte and neuron markers, respectively. GAPDH is set as a loading control.

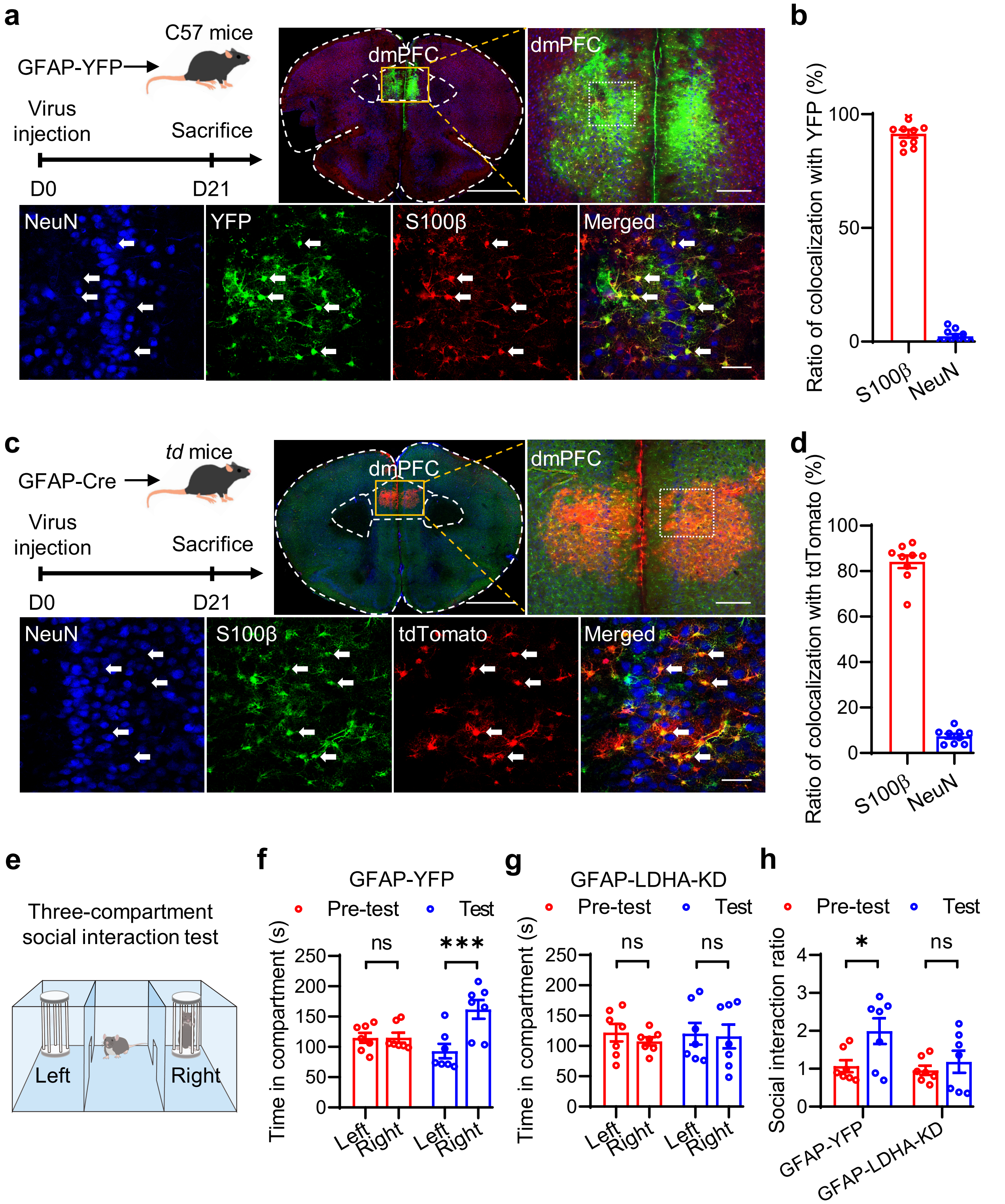

Supplementary Fig. 7. Characterizations of the specificity of GFAP-Cre virus and the effects of LDHA knockdown on social interaction. a GFAP-YFP virus was injected into the dmPFC and brain slices were collected and subjected to staining with astrocytic marker-S100β (red) and Neuronal marker-NeuN (blue). White solid arrows, YFP+ and S100β+. Scale bars: Top left, 2 mm; top right, 200 μm; bottom right, 50 μm. **b** Quantitative analysis. n = 10 slices from 3 mice. For ratio of YFP and S100β, Mean ± SEM = 91.43% ± 1.74%;

For ratio of YFP and NeuN, Mean ± Sem = 2.37% ± 0.91%. **c** GFAP-Cre virus was injected into the dmPFC of *td* mice. Brain slices were collected and subjected to staining with astrocytic marker-S100β (green) and Neuronal marker-NeuN (blue). White solid arrows, tdTomao+ and S100β+. Scale bars: Top left, 2 mm; top right, 200 μm; bottom right, 50 μm. **d** Quantitative analysis. *n* = 9 slices from 3 mice. For ratio of YFP and S100β, mean ± SEM = 84.12% ± 2.76%; For ratio of YFP and NeuN, Mean ± SEM = 7.40% ± 1.06%. **e** Diagram of three-compartment social interaction test. **f** Increased time in the social (right) compartment than in the empty (left) compartment for GFAP-YFP mice. *n* = 7 GFAP-YFP mice. Two-way ANOVA followed by Sidak's post hoc test, $F_{(1,24)} = 9.253$, $p = 0.0056$. Left vs right in pre-test, $p = 0.9997$; left vs right in test, $p = 0.0005$. **g** Comparable time in left and right compartment for GFAP-LDHA-KD mice. *n* = 7 GFAP-LDHA-KD mice. Two-way ANOVA followed by Sidak's post hoc test, $F_{(1,24)} = 0.3706$, $p = 0.5484$. Left vs right in pre-test, $p = 0.77$; left vs right in test, $p = 0.9732$. **h** Not changed social interaction ratio in GFAP-LDHA-KD mice. *n* = 7 mice per group. Paired student's *t*-test, for GFAP-YFP, $t_{(6)} = 2.746$, $p = 0.0335$; for GFAP-LDHA-KD, $t_{(6)} = 0.6847$, $p = 0. 5191$. Data were shown as mean ± SEM. *$p < 0.05$; **$p < 0.01$; ns, no significant difference. Source data are provided as a Source Data file.

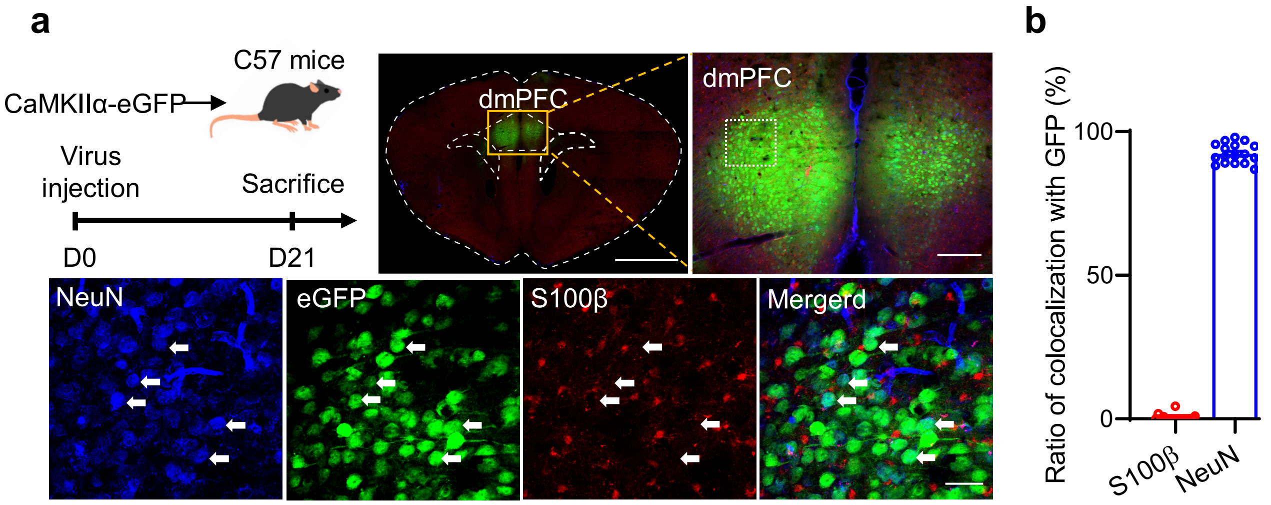

Supplementary Fig. 8. Characterization of the specificity of Camkiiα-eGFP virus. a Camkiiα-ctrl virus was injected into the dmPFC of C57 mice. 21 days later, brain slices were collected and subjected to staining with astrocytic marker-S100β (red) and Neuronal marker-NeuN (blue). White solid arrows denote GFP+ and NeuN+ cells. Scale bars: Top left, 2 mm; top right, 200 μm; bottom right, 50 μm. **b** Quantitative analysis of co-staining ratios. *n* = 17 slices from 3 mice. For ratio of GFP and NeuN, mean ± SEM = 92.4% ± 0.84%; For ratio of GFP and S100β, Mean ± SEM = 0.47% ± 0.28%. Data were shown as mean ± SEM. Source data are provided as a Source Data file.

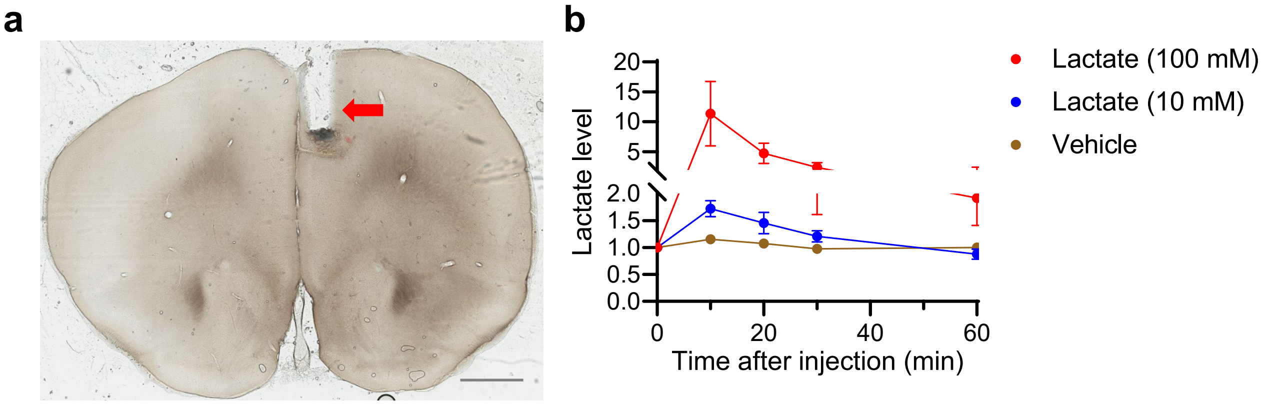

Supplementary Fig. 9. Exhibition of lactate infusion site and characterization of the effect of infusion of lactate on extracellular lactate level in the dmPFC. a Representative image showing a track for cannula implantation in the dmPFC region. Red arrow denotes the cannula track. Scale bar, 1 mm. **b** Infusion of exogenous lactate increases extracellular lactate level in a dose and time dependent manner. L-Lactate (10 mM, 1 μl; 100 mM, 1 μl) or vehicle (1 μl) were infused at a flow rate of 0.2 μl/min. Microdialysis sample was collected every 10 min. n = 3 mice. Data were shown as mean ± SEM. Source data are provided as a Source Data file.

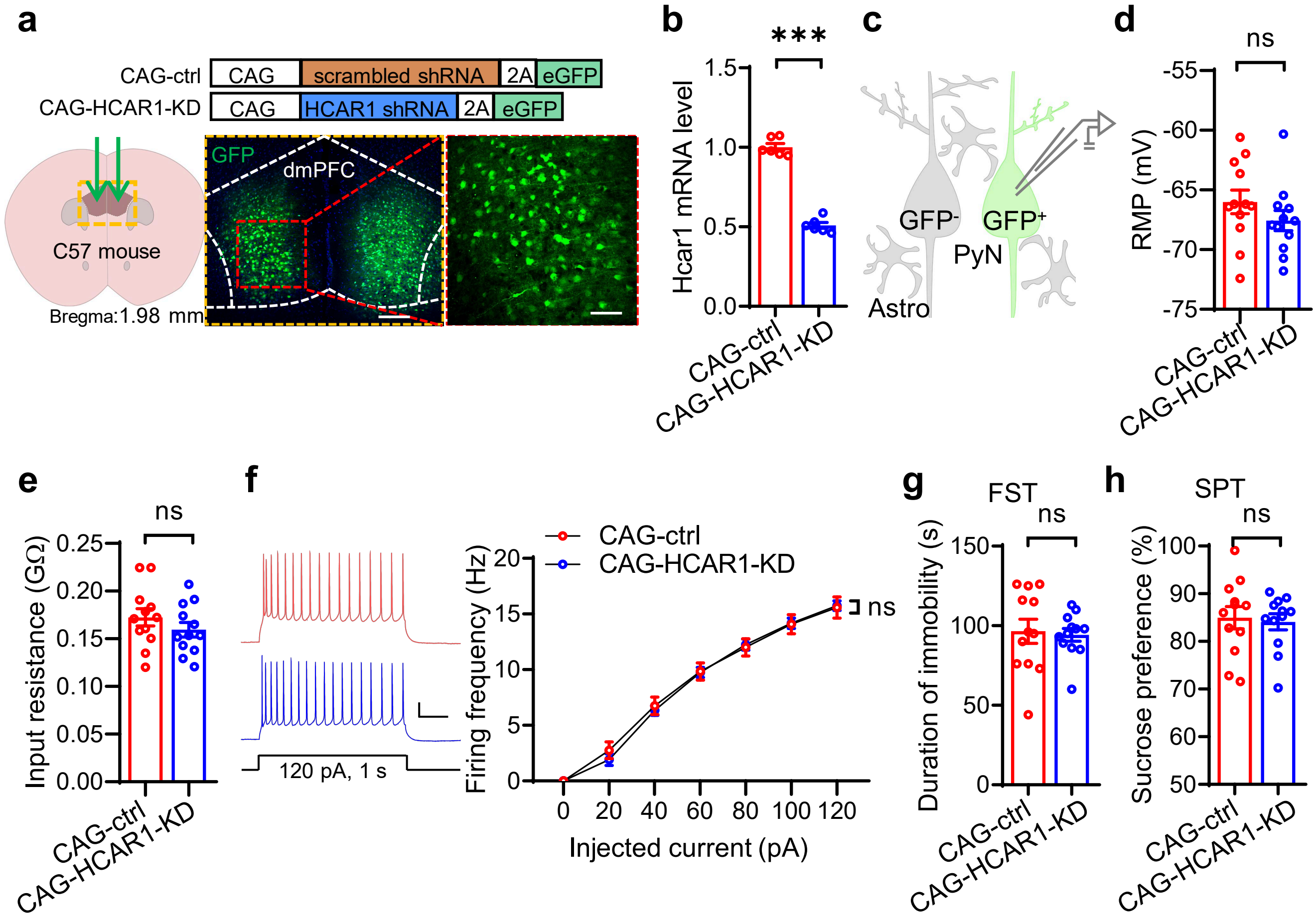

Supplementary Fig. 10. Characterization of the effects of HCAR1 knockdown in the dmPFC on neuronal excitability and depressive-like behaviors. a Top, schematics of AAV vectors that drive expressions of scrambled shRNA (CAG-ctrl) or HCAR1-shRNA (CAG-HCAR1-KD) under the control of a *CAG* promoter. Bottom, illustration of bilateral viral injections in dmPFC region. Scale bars: left, 100 μm; right, 50 μm. **b** RT-PCR result showing that *Hcar1* mRNA level was reduced in dmPFC of CAG-HCAR1-KD mice. *n* = 6 mice per group. Student's *t*-test, $t_{(10)}$ = 16.76, *p* = 0.0000; **c** Schematic diagram of recording of GFP+ neurons in dmPFC region. PyN, pyramidal neuron; Astro, astrocyte. **d** Similar resting membrane potential of neurons in dmPFC. *n* = 12 neurons from 3 mice per group. Student's *t*-test, $t_{(22)}$ = 1.195, *p* = 0.2448. **e** Input resistance of neurons in dmPFC was not changed by HCAR1 knockdown. *n* = 12 neurons from 3 mice per group. Student's *t*-test, $t_{(22)}$ = 1.097, *p* = 0.2843. **f** Not changed neuronal excitability. Left, representative firing traces in response to current injection at an intensity of 120 pA. Scale bars: 0.2 s, 20 mV. Right, quantitative

data. n = 12 neurons from 3 mice per group. Two-way ANOVA, gene effect, $F_{(1,154)} = 0.1306$, $p = 0.7183$; interaction effect, $F_{(6,154)} = 0.1915$, $p = 0.9788$. **g** Comparable immobility duration in FST test. n = 12 mice per group. Student's t-test, $t_{(22)} = 0.264$, $p = 0.7943$. **h** Comparable sucrose preference in SPT test. n = 12 mice per group. Student's t-test, $t_{(22)} = 0.0153$, $p = 0.9879$. Data were shown as mean ± SEM. ***$p < 0.001$; ns, no significant difference. Source data are provided as a Source Data file.

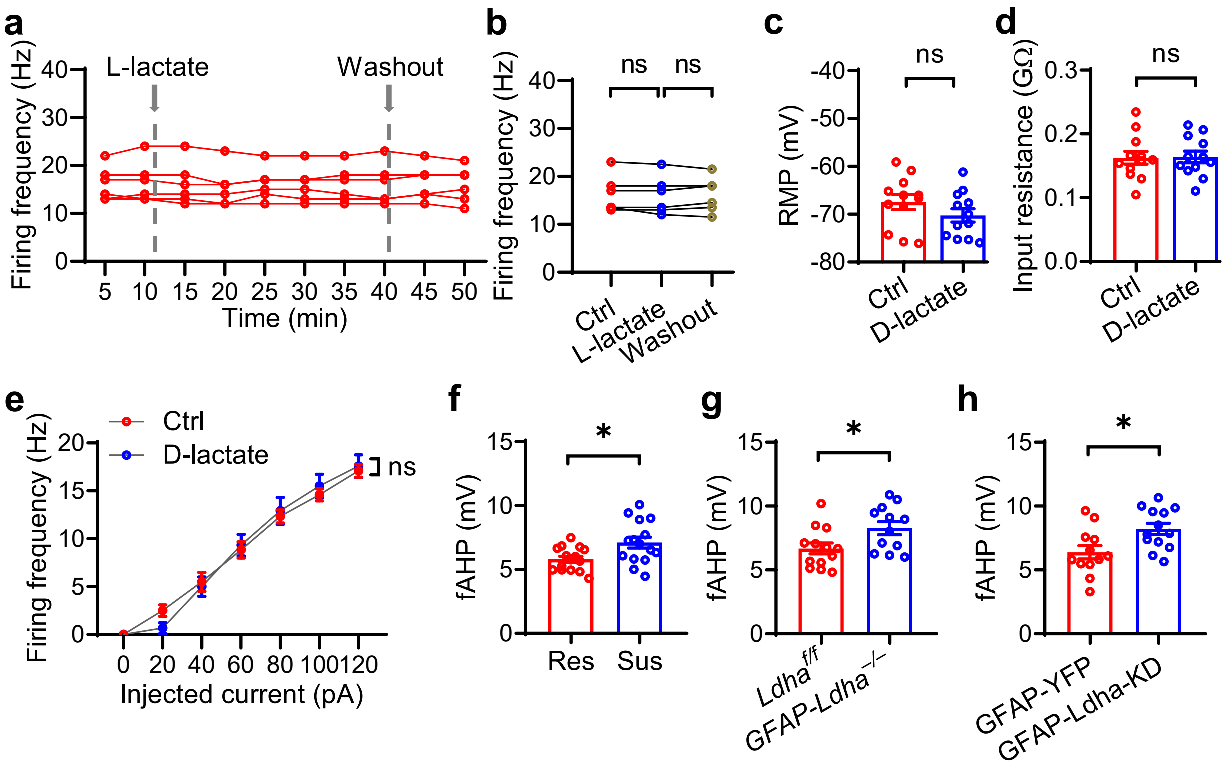

Supplementary Fig. 11. Characterization of the acute effect of L-lactate and chronic effect of D-lactate on neuronal excitability. a Unaltered firing frequencies by acute L-lactate treatment. Firing frequencies in response to current injection at an intensity of 120 pA at different time point from each neuron were individually displayed. The vertical dash line indicates a switch to L-lactate solution and washout solution, respectively. n = 6 neurons from 3 mice. **b** Quantitative analysis of data in (a). Paired t-test, *n* = 6 neurons, L-lactate vs Ctrl, $t_{(5)}$ = 1.348, *p* = 0.2354; Washout vs L-lactate, $t_{(5)}$ = 0.5, *p* = 0.6383. **c** Little effect of D-lactate on resting membrane potential of pyramidal neurons in the dmPFC. *n* = 12 neurons from 4 male C57 mice for each group. Student's *t*-test, $t_{(22)}$ = 1.333, *p* = 0.1963. **d** Little effect of D-lactate on input resistance of pyramidal neurons in the dmPFC. *n* = 12 neurons from 4 male C57 mice for each group. Student's *t*-test, $t_{(22)}$ = 0.095, *p* = 0.9251. **e** Not changed neuronal excitability by D-lactate treatment. *n* = 12 neurons from 4 mice per group. Two-way ANOVA, main effect, $F_{(1,154)}$ = 0.0026, *p* = 0.9596. **f** Increased fAHP amplitude of pyramidal neurons in dmPFC of Sus mice compared with that in Res mice. *n* = 15 neurons from 4 mice per group. Student's *t*-test, $t_{(28)}$ = 2.674, *p* = 0.0124. **g** Increased fAHP

amplitude of pyramidal neurons in dmPFC of *GFAP-Ldha*$^{-/-}$ mice compared with that in *Ldha*$^{f/f}$ mice. n = 13 neurons from 4 male in *Ldha*$^{f/f}$ mice and n = 12 neurons from 4 male *GFAP-Ldha*$^{-/-}$ mice. Student's *t*-test, $t_{(23)} = 2.387$, $p = 0.0256$. **h** Increased fAHP amplitude of pyramidal neurons in dmPFC of GFAP-Ldha-KD mice compared with that in GFAP-YFP mice. n = 12 neurons from 4 male in GFAP-YFP mice and n = 13 neurons from 4 male GFAP-Ldha-KD mice. Student's *t*-test, $t_{(23)} = 2.693$, $p = 0.013$. Data were shown as mean ± SEM. *$p < 0.05$; ns, no significant difference. Source data are provided as a Source Data file.

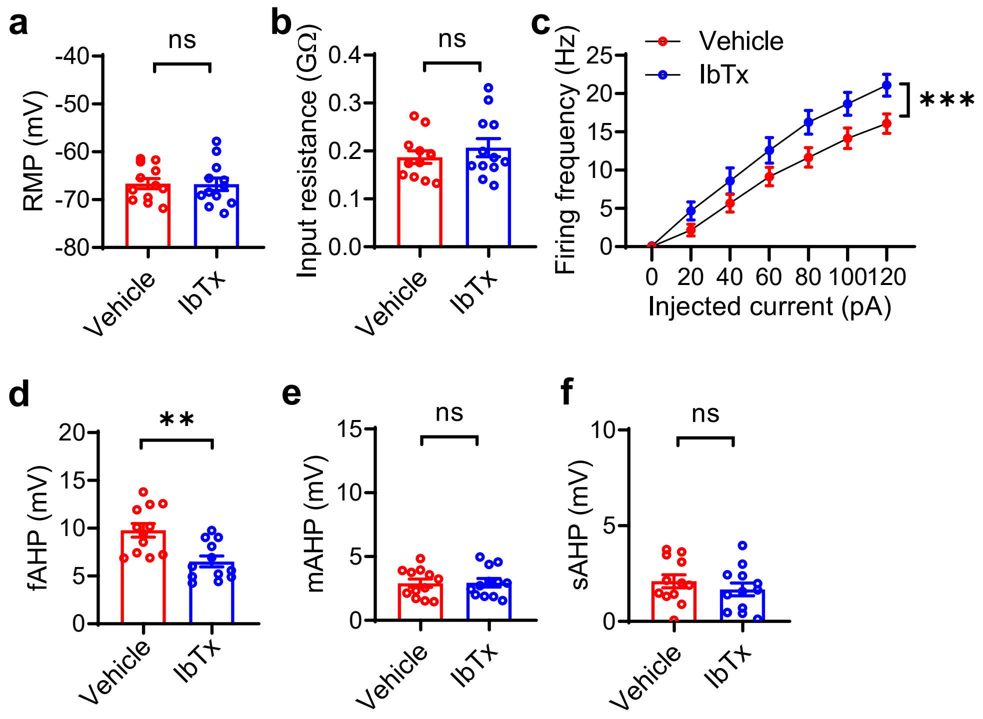

Supplementary Fig. 12. Characterization of the effects of ibtx on neuronal excitability.

a Limited effect of ibtx on resting membrane potential. *n* = 12 neurons from 4 male C57 mice for vehicle and ibtx group, respectively. Student's *t*-test, $t_{(22)}$ = 0.0869, *p* = 0.9316. **b** Limited effect of ibtx on input resistance. *n* = 12 neurons from 4 male C57 mice for vehicle and ibtx group, respectively. Student's *t*-test, $t_{(22)}$ = 0.8701, *p* = 0.3936. **c** Increased neuronal excitability by ibtx treatment. Left, representative firing traces in response to current injection at an intensity of 120 pA. Scale bars: 0.2 s, 20 mV. Right, quantitative data. *n* = 12 neurons from 4 male C57 mice for vehicle and ibtx group, respectively. Two-way ANOVA, gene effect, $F_{(1,154)}$ = 24.07, *p* = 0.0000. **d** Reduced fAHP of pyramidal neurons in dmPFC by ibtx treatment. Left, representative firing traces. Scale bars: 10 ms, 20 mV. Right, quantitative data. *n* = 12 neurons from 4 male C57 mice for vehicle and ibtx group, respectively. Student's *t*-test, $t_{(22)}$ = 3.587, *p* = 0.0016. **e** Not changed mAHP of pyramidal neurons in dmPFC by ibtx treatment. *n* = 12 neurons from 4 male C57 mice for vehicle and ibtx group, respectively. Student's *t*-test, $t_{(22)}$ = 0.0817, *p* = 0.9357. **f** Limited effect of ibtx on sAHP of pyramidal neurons in dmPFC. *n* = 12 neurons from 4 male C57 mice for vehicle and ibtx group, respectively. Student's *t*-test, $t_{(22)}$ = 0.8948, *p* = 0.3806. Data were shown as

mean ± SEM. *$p < 0.05$; ***$p < 0.001$; ns, no significant difference. Source data are provided as a Source Data file.

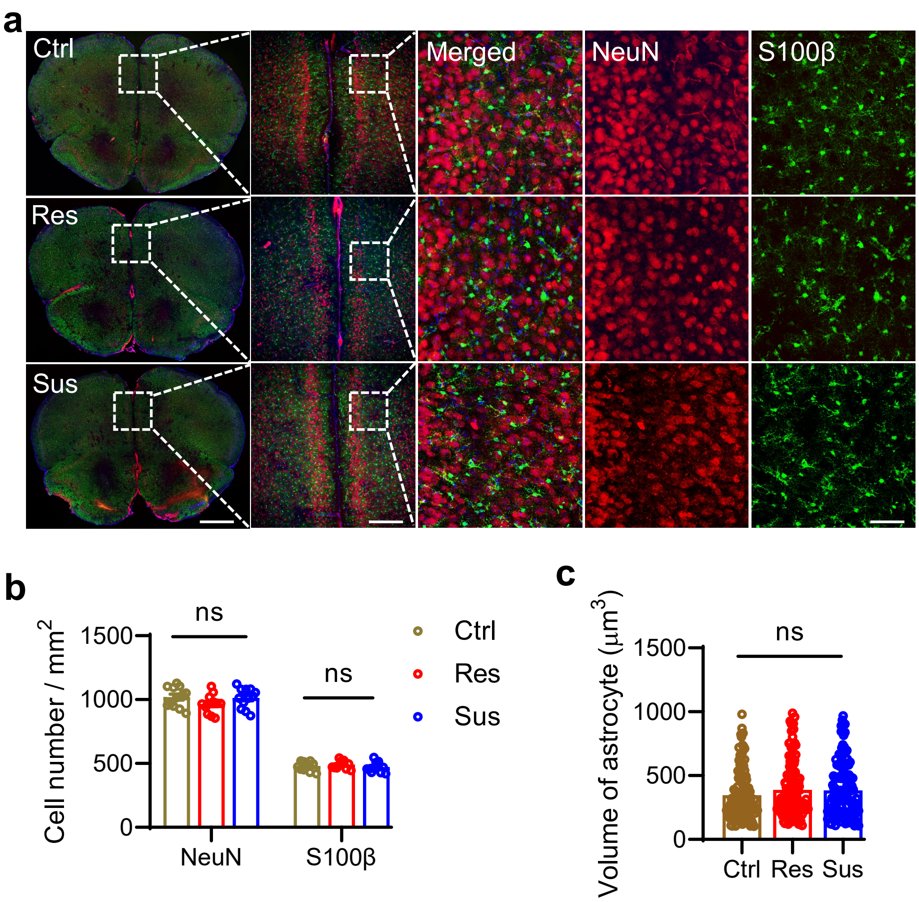

Supplementary Fig. 13. Characterization of cell numbers and volume of astrocytes in the dmPFC of CSDS model. a Representative immunostaining images. Brain slices containing dmPFC from different groups of CSDS model were co-stained with neuronal marker-NeuN (Red) and astrocytic marker-S100β (green), respectively. Dotted squares were enlarged and placed on the right. Scale bars: left, 500 μm, middle, 200 μm, right, 40 μm. **b** Comparable numbers of neuron and astrocyte among the three groups. *n* = 12 slices from 3 mice for each group. One-way ANOVA, for NeuN, $F_{(2,33)} = 1.69$, $p = 0.2001$; for S100β, $F_{(2,33)} = 0.8623$, $p = 0.4315$. **c** Similar volume of astrocytes among the three groups. *n* = 123 S100β+ cells from 3 Ctrl mice; *n* = 127 S100β^{+} cells from 3 Res mice; *n* = 136 S100β^{+} cells from 3 Sus mice. One-way ANOVA, $F_{(2,383)} = 1.551$, $p = 0.2143$. Data were shown as mean ± SEM. ns, no significant difference. Source data are provided as a Source Data file.

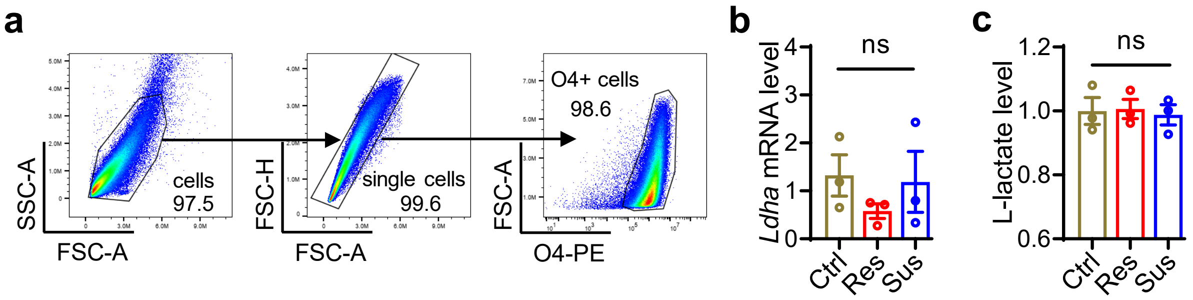

Supplementary Fig. 14. Characterizations of *Ldha* mRNA and lactate levels in oligodendrocytes purified from the dmPFC of CSDS mice. **a** O4-positive cell gating strategy. 98.6% of all viable isolated cells are O4-positive. **b** Not changed *Ldha* mRNA levels. n = 3 samples from 12 mice for each group. One-way ANOVA, $F_{(2,6)} = 0.7676$, p = 0.5466. **c** Not changed lactate levels. n = 3 samples from 12 mice for each group. One-way ANOVA, $F_{(2,6)} = 0.0735$, p = 0.93. Data were shown as mean ± SEM. ns, no significant difference. Source data are provided as a Source Data file.
